# Climatic and drought characteristics in the loess hilly-gully region of China from 1957 to 2014

**DOI:** 10.1371/journal.pone.0178701

**Published:** 2017-06-08

**Authors:** Xingkai Zhao, Zengyao Li, Qingke Zhu, Danhong Zhu, Huifang Liu

**Affiliations:** 1 School of Soil and Water Conservation, Beijing Forestry University, Beijing, China; 2 Planning and Design Institute of Forest Products Industry, State Forestry Administration, Beijing, China; 3 State Key Laboratory of Simulation and Regulation of Water Cyclein River Basin, China Institute of Water Resources and Hydropower Research, Beijing, China; Pacific Northwest National Laboratory, UNITED STATES

## Abstract

The loess hilly-gully region is a focus region of the “Grain for Green” program in China. Drought is the main problem in the study region. Precipitation and temperature are two indicators that directly characterize climatic drought. A thorough analysis of the precipitation, temperature and drought characteristics of the loess hilly-gully region can clarify the current water and heat conditions in the region to improve regional water resource management and provide a reliable reference for effectively improving water use efficiency. In this study, we fully analyzed the precipitation and temperature characteristics at 11 representative synoptic stations in the loess hilly-gully region over the period from 1957 to 2014 using a combination of trend-free pre-whitening, linear trend estimation, Spearman’s rho test, the Mann–Kendall (M-K) trend and abrupt change tests and wavelet analysis. The standardized precipitation evapotranspiration index was calculated and analyzed on different time scales. The following conclusions were drawn: (1) There were significant spatial differences and inter-annual variations in precipitation at the 11 synoptic stations in the study area between 1957 and 2014; the precipitation consistently decreased with fluctuations, and the extent of the decrease varied from a maximum of 17.74 mm/decade to a minimum of 2.92 mm/decade. Except for the downward trends of the autumn and winter mean temperatures at Hequ, the seasonal and annual mean temperatures at the stations showed upward trends, including highly significant upward trends. (2) Alternating drought and wetness occurred in the study area; the wet period mainly appeared in the 1960s, and the main dry period lasted from the late 20th century to 2012. There were fewer dry and wet years than normal years; however, the study area still showed a drying trend, and the severity of the drought was increasing. (3) The annual precipitation and annual mean temperature showed marked cyclical fluctuations at each synoptic station, and the first primary cycle was approximately 28 years. The seasonal precipitation and seasonal temperature showed different cycle lengths; the seasonal cycles of precipitation for spring, summer, autumn and winter were 10, 28, 10 and 26 years long, respectively, and the cycles of the temperature fluctuations for all four seasons were approximately 28 years long.

## Introduction

Precipitation and temperature are two indicators that directly characterize climatic drought. Drought is a natural disaster that is common worldwide and a natural phenomenon in which the amount of water available is insufficient for a long period. The Loess Plateau in China is a serious region of global drought [1]. It is also one of the regions suffering the most serious soil erosion in China and even in the world. The Loess Plateau has long been plagued by soil erosion [2], where the agricultural production, animal husbandry, and ecological environment strongly depend on climatic conditions [3, 4]. To control soil erosion and improve the environment, the Chinese government issued the “Grain for Green” policy in 1999 for vegetation restoration, and the loess hilly-gully region [5] was one of the ten important regions under this policy. Soil moisture is a major limiting factor for plant growth and vegetation construction in arid and semiarid regions [6]. In the Loess Plateau, atmospheric precipitation is the only way to recharge soil moisture, but the atmospheric temperature affects the evapotranspiration of water. It is necessary to analyze the regions with the same and precise geographical divisions in order to get a more accurate conclusion. Therefore, in the context of global climate change, investigating the characteristics of regional drought and wetness plays a relatively strong supporting role in understanding the dynamic changes in soil moisture and it is helpful to improve the regional water resources planning and management, improve water use efficiency.

For studies that analyze climate drought and wetness, the commonly selected indices include the Palmer drought severity index (PDSI) [7], the standardized precipitation index (SPI) [8] and the standardized precipitation evapotranspiration index (SPEI) [9]. The PDSI [10], an indicator of drought and wetness, is based on a water balance equation. The PDSI is a milestone indicator in drought analyses, but the procedure for calculating it is complex, and the parameter is strongly regional. The SPI [11] is a drought index recommended by the World Meteorological Organization. The procedure for calculating the SPI is only based on precipitation data, and it gives no consideration to other variables (e.g., evapotranspiration and wind speed) that may affect the drought. To improve the SPI, Sergio M. Vicente-Serrano proposed the SPEI [12]. The characteristics of the precipitation on the Loess Plateau in China have been reported, normally using the SPI [13, 14]. However, no study has been conducted based on the SPEI to fully analyze the precipitation and temperature in the loess hilly-gully region of the Loess Plateau. A thorough analysis of the precipitation, temperature and drought characteristics in the loess hilly-gully region can clarify the current water and heat conditions in the region to improve regional water resource management and provide a reliable reference for effectively improving water use efficiency and further improving the efficiency of vegetation restoration. In the present study, we analyzed the precipitation and temperature data from 11 representative synoptic stations within the loess hilly-gully region over the period from 1957 to 2014. The objectives of the study were (1) to determine the precipitation and temperature changes at the synoptic stations on monthly, seasonal and annual time scales using a combination of trend-free pre-whitening, linear trend estimation, Spearman’s rho test and the Mann–Kendall (M-K) trend and abrupt change tests, (2) to interpret the SPEI on spatial and temporal scales and clarify the drought and wetness conditions and the trends of changes in the study area over the period from 1957 to 2014 and (3) to identify the cycle of precipitation and temperature changes using wavelet analysis.

## Material and methods

### Study area and data source

**Fig 1:**
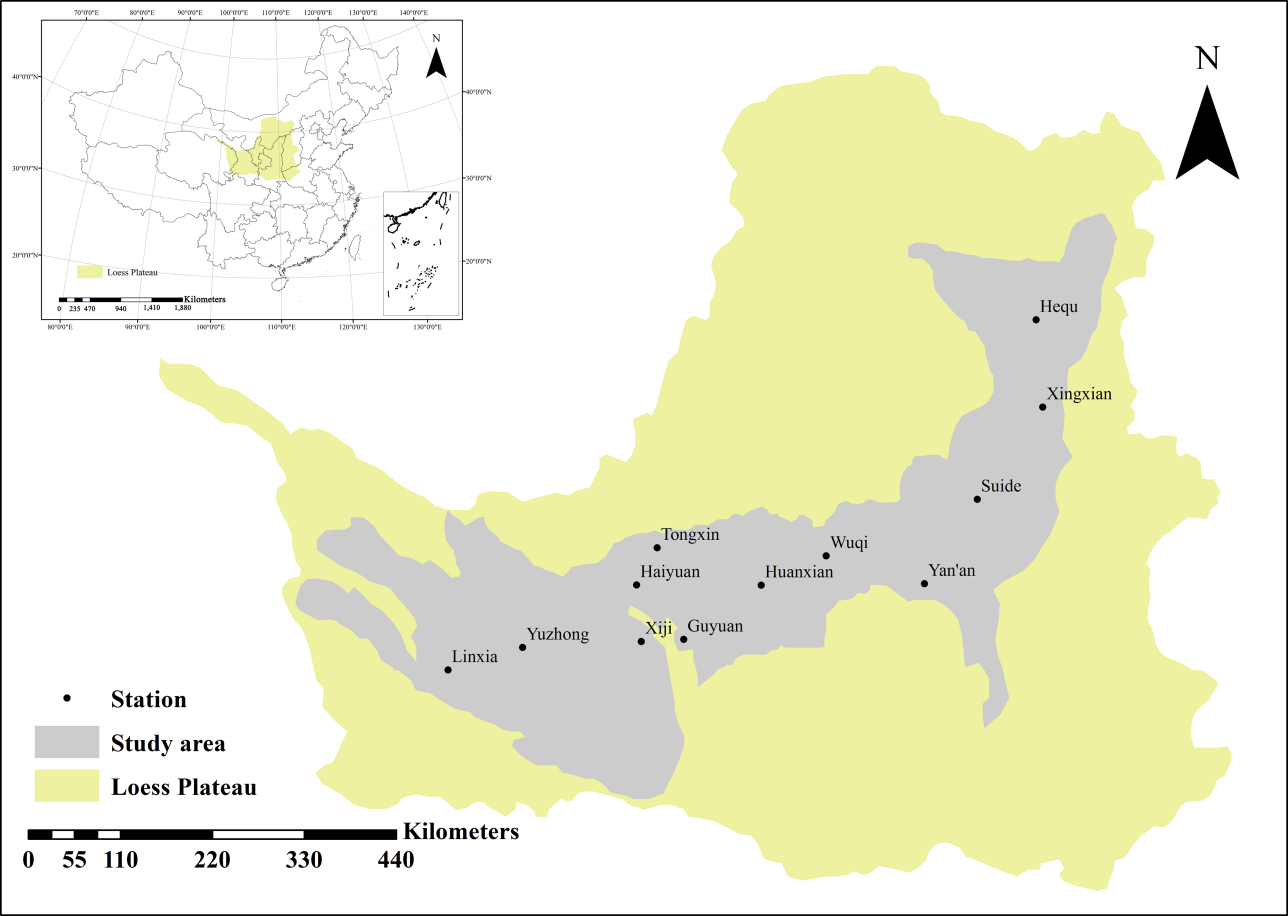
The spatial distribution of the 11 synoptic stations.

The loess hilly-gully region is the remaining land between gullies after long-term gully segmentation and runoff erosion of loess tableland. This part of the region is most extensively distributed on the Loess Plateau. It covers an area of 211,000 km^2^ across seven provinces (autonomous regions) in China: Henan, Shanxi, Shaanxi, Inner Mongolia, Ningxia, Gansu and Qinghai. The loess hilly-gully region has become the main body of the Loess Plateau landscape, accounting for approximately 33% of the total land area of the plateau region. It is one of the major regions under the “Grain for Green” policy in China (the southwest alpine gorge region, the Sichuan-Chongqing-Hubei-Hunan mountainous and hilly region, the low mountainous and hilly region near the middle and lower Yangtze River, the Yunnan-Guizhou Plateau region, the Hainan-Guangxi hilly and mountainous region, the alpine steppe and meadow region at the origin of the Yangtze and Yellow Rivers, the Xinjiang arid desert region, the loess hilly-gully region, the north arid and semiarid region and the northeast mountainous and sandy region). The study area is located in the main part of the loess hilly-gully region (Fig 1).

The meteorological data used in this study were obtained from the China Meteorological Data Sharing Service Network (http://data.cma.cn/). To ensure the synchronization of the meteorological data and a long time series of observations at each synoptic station, stations with missing data points were excluded, and a total of 11 representative stations were chosen in the present study (Table 1). The precipitation and temperature data from 1957 to 2014 were used.

**Table 1:** Geographical descriptions of the synoptic stations used in this study.

Station name	Longitude (E)	Latitude (N)	Elevation (m a.s.l)	Province (autonomous regions)
**Lintao**	103°51′	35°21′	1893.8	Gansu
**Linxia**	103°11′	35°35′	1917.2	Gansu
**Huanxian**	107°18′	36°35′	1255.6	Gansu
**Yuzhong**	104°09′	35°52′	1874.4	Gansu
**Guyuan**	106°16′	36°00′	1753.0	Ningxia
**Tongxin**	105°54′	36°58′	1339.3	Ningxia
**Hequ**	111°09′	39°23′	861.5	Shanxi
**Xingxian**	111°08′	38°28′	1012.6	Shanxi
**Suide**	110°13′	37°30′	929.7	Shaanxi
**Wuqi**	108°10′	36°55′	1331.1	Shaanxi
**Yan’an**	109°30′	36°36′	958.5	Shaanxi

### Study methods

#### Standardized precipitation evapotranspiration index

Sergio M. Vicente-Serrano proposed the SPEI to improve the SPI, which gives no consideration to temperature. When calculating the SPEI, the monthly precipitation and the monthly mean temperature are taken as the input variables. The SPEI is obtained by calculating the difference between the monthly precipitation and the potential evapotranspiration, followed by normalization [12]. The SPEI not only retains the characteristics of the PDSI concerning the temperature sensitivity of evapotranspiration but also possesses the SPI’s advantages of being simple to calculate and suitable for multi-scale and multi-space comparisons. The SPEI can be calculated as follows [12]:

a) First, following the Thornthwaite [15] method, the monthly *PET* (mm) is obtained from

$$PET=16\cdot \left(\frac{N}{12}\right)\;\left(\frac{NDM}{30}\right)\;{\left(\frac{10T}{I}\right)}^{m},$$ (1)

where *m* = 6.75 × 10^−7^*I*^3^ − 7.71 × 10^−5^*I*^2^ + 1.79 × 10^−2^*I* + 0.492; *T*(°C) is the monthly mean temperature; *N* is the mean number of hours of sunshine; *NDM* is the number of days of the month; and *I* is a heat index, which is calculated using

$$I=\overset{12}{\underset{i=1}{\sum }}{\left(\frac{T_{i}}{5}\right)}^{1.514}.$$ (2)

b) Then, the difference between the precipitation, *P*, and the *PET* for month *i* is calculated using

$$D_{i}=P_{i}-PET_{i}.$$ (3)

c) Next, the different time scales of the cumulative moisture deficit ullage sequence, *X*, are identified using

$$X_{k}^{n}=\overset{k-1}{\underset{i=0}{\sum }}\left(P_{n-i}-PET_{n-i}\right),\;n\geq k,$$ (4)

where *n* is the number of time series samples and *k* is the time scale.

d) The probability density function of a three-parameter log-logistic distributed variable is expressed as

$$f\left(x\right)=\frac{\beta }{\alpha }{\left(\frac{x-\gamma }{\alpha }\right)}^{\beta -1}\;{\left[1+{\left(\frac{x-\gamma }{\alpha }\right)}^{\beta }\right]}^{-2},$$ (5)

where *α*, *β* and *γ* are scale, shape and origin parameters, respectively, which can be obtained using the following:

$$\alpha =\frac{\left(w_{0}-2w_{1}\right)\beta }{\Gamma \left(1+\frac{1}{\beta }\right)\Gamma \left(1-\frac{1}{\beta }\right)},$$ (6)

$$\beta =\frac{2w_{1}-w_{0}}{\left(6w_{1}-w_{0}-6w_{2}\right)},$$ (7)

and

$$\gamma =w_{0}-\alpha \Gamma \left(1+\frac{1}{\beta }\right)\Gamma \left(1-\frac{1}{\beta }\right).$$ (8)

Then, *w*_0_, *w*_1_ and *w*_2_ can be calculated using

$$w_{s}=\frac{1}{n}\overset{n}{\underset{l=1}{\sum }}{\left(1-\frac{l-0.35}{n}\right)}^{s}X_{l},$$ (9)

where *w*_*s*_ is a probability-weighted moment, *s* = 0, 1, 2; *l* is the index in the cumulative moisture deficit ullage sequence arranged in ascending order and *Γ*(*β*) is the gamma function of *β*. The probability distribution function of the *D* series, according to the log-logistic distribution, is given by

$$F\left(x\right)={\left[1+{\left(\frac{\alpha }{x-\gamma }\right)}^{\beta }\right]}^{-1}.$$ (10)

e) The SPEI can be obtained as the standardized values of *F*(*x*),

$$SPEI=\{\begin{array}{c} w-\frac{c_{0}+c_{1}w+c_{2}w^{2}}{1+d_{1}w+d_{2}w^{2}+d_{3}w^{3}},w=\sqrt{-2\ln\left(1-F\left(x\right)\right)},for\;1-F\left(x\right)\leq 0.5\\ \frac{c_{0}+c_{1}w+c_{2}w^{2}}{1+d_{1}w+d_{2}w^{2}+d_{3}w^{3}}-w,w=\sqrt{-2\ln\left(F\left(x\right)\right)},for\;1-F\left(x\right)>0.5 \end{array},$$ (11)

where the constants are *c*_0_ = 2.515517, *c*_1_ = 0.802853, *c*_2_ = 0.010328, *d*_1_ = 1.432788, *d*_2_ = 0.189269 and *d*_3_ = 0.001308.

For the SPEI, the drought threshold has not been defined. In this study, we combined the drought grade classification methods and criteria of the SPI [11], the Climate Prediction Center [16], and the China National Meteorological Center [17]. The tentative range of the SPEI and the drought-wetness grade classification criteria are shown in Table 2.

**Table 2:** Drought categories.

SPEI	Classification
**≥ 2.0**	Extremely wet
**1.5–1.99**	Very wet
**1.0–1.49**	Moderately wet
**-0.99–0.99**	Near normal
**-1.49 –-1.0**	Moderately dry
**-1.99 –-1.5**	Severely dry
**≤ -2**	Extremely dry

#### Trend-free pre-whitening

The purpose of trend-free pre-whitening (TFPW) is to eliminate the influence of autocorrelation of the data sequence on the change trend [18]. The calculation steps are as follows:

a) The slope (β) of a trend in the sample data is estimated using the approach proposed by Theil [19] and Sen [20]. The original sample data, *X*_*t*_, are normalized by dividing each value by the sample mean, E (*X*_*t*_), prior to the trend analysis. After this treatment, the mean of the new data points, *x*_*t*_, is unity, and the properties of the original sample data remained unchanged. If the slope is almost zero, then it is not necessary to continue to complete the trend analysis. If it differs from zero, then it is assumed to be linear, and the sample data are de-trended using

$$X_{t}^{\prime }=X_{t}-T_{t}=X_{t}-\beta \cdot t,$$ (12)

where the slope, *β*, is calculated using

$$\beta =\mathrm{median}\frac{x_{j}-x_{i}}{j-i}\;\forall j>i.$$ (13)

b) The lag-1 serial correlation coefficient of the sample data, *x*_*i*_, (designated *R*_*h*_) is expressed by

$$R_{h}=\frac{\frac{1}{n}\sum_{i=1}^{n-1}(x_{i}-\mu \left(x_{i}\right))\cdot \left(x_{i+1}-\mu \left(x_{i}\right)\right)}{\frac{1}{n}\sum_{i=1}^{n}{\left(x_{i}-\mu \left(x_{i}\right)\right)}^{2}}$$ (14)

$$\mu \left(x_{i}\right)=\frac{1}{n}\overset{n}{\underset{i=1}{\sum }}x_{i}.$$ (15)

For the two-sided test, *R*_*h*_ is computed using the following equation at the 95% significance level [21]:

$$\frac{-1-1.96\sqrt{n-2}}{n-1}<R_{h}\left(95\%\right)<\frac{-1+1.96\sqrt{n-2}}{n-1}.$$ (16)

Where n is the sample size.

The lag-1 serial correlation coefficient of the detrended series, $X_{t}^{\prime }$, is *r*_1_. If *r*_1_ falls within the range calculated using Eq (16), then the sample data are considered serially independent and a linear trend estimation, Spearman’s rho test and the Mann–Kendall tests are applied directly to the $X_{t}^{\prime }$. Otherwise, the calculation is continued, and sample data, $Y_{t}^{\prime }$, are obtained using

$$Y_{t}^{\prime }=X_{t}^{\prime }-r_{1}\cdot X_{t-1}^{\prime }.$$ (17)

This procedure for pre-whitening the detrended series is referred to as TFPW. The series that remains after TFPW should be independent.

c) The identified trend (*T*_*t*_) and the residual, $Y_{t}^{\prime }$, are combined as follows:

$$Y_{t}=Y_{t}^{\prime }+T_{t}.$$ (18)

The blended series (*Y*_*t*_) consists of the trend and noise and is no longer influenced by serial correlation. Then, the linear trend estimation, Spearman’s rho test and the Mann–Kendall tests are applied to the blended series to assess the significance of the trend.

#### Linear trend estimation

*x*_*i*_ denotes a climate variable with a sample size of *n*, and *t*_*i*_ denotes the time corresponding to *x*_*i*_. A linear regression equation is established between *x*_*i*_ and *t*_*i*_,

$$x_{i}=a+bt_{i},\;i=1,2,\ldots ,n.$$ (19)

The above equation can be regarded as a special case and the simplest form of linear regression, wherein a is the regression constant and *b* is the regression coefficient; *a* and *b* can be estimated using the least squares method. The regression coefficient, *b*, indicates the trend of the climate variable. That is, *b* > 0 indicates that climate variable *x* increases with time, and *b* < 0 indicates the opposite trend. The value of *b* reflects the rate of increase or decrease, namely, the degree of the tendency. In general, *b* (*b* × 10) indicates the trend rate [units: mm/year or °C/year (mm/decade or °C/decade)], which can be used to quantitatively analyze linear trends of changes in climate elements.

#### Mann–Kendall trend test

In the M-K [22, 23] trend test, the null hypothesis, H_0_, is that the time series data (*x*_*1*_, *x*_*2*_, …, *x*_*n*_) comprise a sample of *n* independent and identically distributed random variables. The alternative hypothesis, H_1_, is a bilateral test: for all *k*, *j ≤ n* and *k* ≠ *j*, *x*_*k*_ and *x*_*j*_ are differently distributed. The statistical variable, *S*, is calculated using

$$S=\overset{n-1}{\underset{k=1}{\sum }}\overset{n}{\underset{j=k+1}{\sum }}Sgn\left(x_{j}-x_{k}\right),$$ (20)

where *Sgn*(*x*) is the sign function, whose value is determined as shown in the following equation:

$$Sgn\left(x_{j}-x_{k}\right)=\{\;\begin{array}{c} +1\;x_{j}-x_{k}>0\\ 0\;x_{j}-x_{k}=0.\\ -1\;x_{j}-x_{k}<0 \end{array}$$ (21)

*S* is normally distributed and has a mean of zero. The variance is denoted as *Var* = *n*(*n* − 1)(2*n* + 5)/18; when *n* > 10, the standard normal statistical variable, *Z*, can be calculated as follows:

$$Z=\{\begin{array}{c} \frac{S-1}{\sqrt{Var\left(S\right)}}\;S>0\\ 0\;S=0\\ \frac{S+1}{\sqrt{Var\left(S\right)}}\;S<0 \end{array}.$$ (22)

In the bilateral trend test, if *|Z|* ≥ *Z*_1−(*α*/2)_ at a given confidence level, *α*, the null hypothesis, H_0_, is unacceptable; that is, the time series data show a significant upward or downward trend at the *α* confidence level. If *Z* > 0, the series shows an upward or increasing trend; if *Z* < 0, the series shows a downward or decreasing trend. *|Z|* ≥ 1.28, 1.64 and 2.32 indicate that the data pass the significant trend test at the 90%, 95% and 99% confidence levels, respectively.

#### Mann–Kendall abrupt change test

A rank series is constructed for the time series, *x*, with a sample size of *n* as follows:

$$s_{k}=\overset{k}{\underset{i=1}{\sum }}r_{i}\;\left(k=2,3,\ldots ,n\right).$$ (23)

When *x*_*i*_ > *x*_*j*_, *r*_*i*_ = +1 and when *x*_*i*_ < *x*_*j*_, *r*_*i*_ = 0 (*j* = 1, 2, …, *i)*.

The statistical variable is defined as follows based on the assumption that the time series is independent and random:

$$UF_{K}=\frac{\left[s_{k}-\bar{s_{k}}\right]}{\sqrt{Var\left(s_{k}\right)}}\;\left(k=1,2,\ldots ,n\right),$$ (24)

where UF_1_ = 0 and $\overset{-}{s_{k}}$ and Var(s_*k*_) are the mean and variance of the cumulative number *s*_*k*_, respectively. When *x*_*1*_, *x*_*2*_…, *x*_*n*_ are independent in the same continuous distribution, $\overset{-}{s_{k}}$ and *Var*(*s*_*k*_) can be calculated using

$$\{\begin{array}{c} \bar{s_{k}}=\frac{n\left(n+1\right)}{4}\\ Var\left(s_{k}\right)=\frac{n\left(n-1\right)\left(2n+5\right)}{72} \end{array}.$$ (25)

UF_*i*_, a standard normal distribution, is a series of statistics calculated following the order of the time series, *x*. At a given significance level, *α*, |UF_*i*_| > U_*α*_ indicates a significant trend in the series. The above calculation procedure is repeated in the reverse order of the time series, *x*, and UB_*k*_ = −UF_*k*_ (*k* = *n*, *n*−1, …, 1) with UB_1_ = 0. Then, UF_*k*_ > 0 indicates an upward or increasing trend, UF_*k*_ < 0 indicates a downward or decreasing trend, and exceeding the critical line indicates a significant trend. If the two curves formed by UF_*k*_ and UB_*k*_ intersect and if their intersection appears between the boundary lines, the corresponding time of the intersection is considered the time at which an abrupt change begins.

#### Spearman’s rho test

Spearman’s rho test [24, 25] is a non-parametric test that is commonly used to determine the trends of time series that are not normally distributed. It is expressed by

$$D=1-\frac{\left[6\sum_{i=1}^{n}{\left(R\left(X_{i}\right)-i\right)}^{2}\right]}{n\left(n^{2}-1\right)},$$ (26)

$$Z_{D}=D\sqrt{\frac{n-2}{1-D^{2}}},$$ (27)

where *n* is the length of the time series; *R*(*X*_*i*_) is the rank of the time series at observed value *X*_*i*_ and positive and negative values of *Z*_*D*_ indicate upward and downward trends, respectively. |*Z*_*D*_| > 2.08 rejects the hypothesis that there is no trend at the 5% significance level.

#### Wavelet analysis

Wavelet analysis is considered a breakthrough in Fourier analysis. In climate diagnoses, the extensively used Fourier transform can show the relative contributions of climate series on different scales. The wavelet transform provides the scales of climate series and shows the times at which the changes occur. The latter method is very effective for climate prediction and has been used in climate analysis [26–28].

For a time series *f*(*t*), the wavelet transform is defined as

$$W_{f}\left(a,b\right)=\frac{1}{\sqrt{a}}\int_{-\infty }^{\infty }f\left(t\right)\Psi^{*}\left(\frac{t-b}{a}\right)dt,$$ (28)

where *W*_*f*_(*a*,*b*) is the wavelet coefficient, *a* is the scale factor (which determines the wavelet coefficient), *b* is the shift factor (which reflects changes in the wavelet’s position) and Ψ^*^ is the conjugate of *Ψ*.

In this study, a Morlet wavelet analysis was conducted on the selected time series using Matlab (R2010b) to analyze cyclic changes in the precipitation and temperature in the loess hilly-gully region on different time scales. The continuous wavelet transform is expressed analytically as

$$\Psi \left(x\right)=Ce^{-\frac{x^{2}}{2}\cos\left(5x\right)}.$$ (29)

The wavelet variance can be expressed as

$$W_{p}\left(a\right)=W_{f}{\left(a,b\right)}^{2}.$$ (30)

## Results

### Precipitation analysis

#### Precipitation characteristics

Table 3 shows the characteristics of the monthly precipitation at the 11 synoptic stations in the study area over the period from 1957 to 2014. The monthly mean precipitation ranged from 22.4 to 45.3 mm; the lowest and highest values were found at Tongxin and Yan’an, respectively. The coefficients of variation (CVs) of the monthly precipitation were greater than 100% at the synoptic stations; the highest value (136.37%) occurred at Hequ, and the lowest value (105.34%) occurred at Linxia.

**Table 3:** Statistical parameters of the monthly precipitation time series for the 11 synoptic stations during the period from 1957 to 2014.

Station name	Min (mm)	Max (mm)	Mean (mm)	Standard deviation (mm)	CV (%)	Skewness	Kurtosis
**Lintao**	0.0	355.6	44.1	46.7	105.85	1.49	3.52
**Linxia**	0.0	217.1	42.1	44.4	105.34	1.16	0.81
**Huanxian**	0.0	312.6	36.2	45.2	125.05	2.23	6.86
**Yuzhong**	0.0	207.0	32.2	36.7	114.01	1.61	2.81
**Guyuan**	0.0	324.4	37.9	45.8	120.92	2.03	5.61
**Tongxin**	0.0	207.1	22.4	28.3	126.32	1.95	4.82
**Hequ**	0.0	339.4	34.2	46.7	136.37	2.43	7.78
**Xingxian**	0.0	349.3	40.9	50.2	122.87	1.98	4.63
**Suide**	0.0	365.8	37.7	48.2	127.74	2.22	6.75
**Wuqi**	0.0	317.6	39.3	47.8	121.60	1.79	3.63
**Yan’an**	0.0	568.0	45.3	55.2	121.91	2.52	12.92

CV: Coefficient of variation.

#### Autocorrelation analysis of the precipitation series

The serial correlation coefficient can improve the verification of the independence of the precipitation time series. In this study, the lag-1 serial correlation coefficients (r_1_) at different scales (monthly, seasonal and annual) at each station were calculated using Eq 14 (Table 4). For precipitation time series, if the value lag-1∈ (-0.2748, 0.2398), based on Eq 16 (n = 58), then, the null hypothesis that the time series data show no autocorrelation was accepted. The data in Table 4 show that there were very few autocorrelations on monthly, seasonal and annual time scales among the meteorological stations in the study area; the values fell between -0.2748 and 0.2398 after the time series of the autocorrelation precipitation data were corrected (the cells in gray in the table) using TFPW. Autocorrelations in the original precipitation data were completely eliminated, allowing linear trend estimation, Spearman’s rho test and the M-K test to be applied [29].

**Table 4:** Lag-1 serial correlation coefficients of precipitation on different scales.

Station	Jan.	Feb.	Mar.	Apr.	May.	Jun.	Jul.	Aug.	Sep.	Oct.	Nov.	Dec.	Spring	Summer	Autumn	Winter	Annual
**Lintao**	0.074	0.089	-0.241	-0.012	-0.013	0.070	0.106	-0.079	0.049	0.087	0.042	-0.127	-0.075	0.031	0.230	0.186	0.082
**Linxia**	-0.041	0.102	-0.127	0.149	0.109	0.095	0.071	-0.158	0.035	0.218	0.092	-0.136	0.021	-0.200	0.176	0.178	0.038
**Huanxian**	-0.051	0.042	0.070	0.144	-0.077	0.108	-0.035	-0.089	0.211	-0.010	-0.092	0.126	0.048	-0.194	-0.039	0.072	-0.089
**Yuzhong**	-0.046	0.144	-0.239	-0.158	-0.013	-0.001	0.177	-0.043	-0.135	0.009	-0.015	-0.138	-0.128	-0.105	0.085	-0.001	-0.113
**Guyuan**	-0.036	0.214	-0.036	0.064	0.114	-0.042	0.175	-0.038	0.100	-0.064	-0.013	0.011	0.027	-0.178	-0.022	0.057	-0.075
**Tongxin**	-0.128	0.063	-0.012	-0.178	0.051	-0.059	0.158	-0.059	0.053	-0.162	0.009	0.034	0.079	-0.074	0.087	0.211	-0.073
**Hequ**	0.199	-0.062	0.109	0.116	0.154	-0.121	-0.159	-0.019	0.034	-0.055	0.099	-0.018	0.214	-0.157	0.055	0.202	-0.156
**Xingxian**	0.135	-0.155	0.002	0.006	0.004	0.086	0.005	0.023	-0.047	-0.136	0.036	-0.098	0.058	-0.208	0.000	-0.165	-0.172
**Suide**	-0.090	-0.121	0.035	0.076	-0.105	0.015	0.001	0.192	-0.057	-0.161	-0.137	-0.073	0.066	0.027	-0.075	-0.086	-0.010
**Wuqi**	-0.015	-0.122	0.044	0.041	-0.067	0.005	-0.064	-0.011	0.137	-0.15	0.078	0.126	0.012	-0.196	0.062	-0.010	-0.165
**Yan’an**	-0.030	-0.091	0.187	-0.062	-0.067	-0.162	-0.062	-0.075	0.154	-0.142	-0.024	0.051	-0.030	-0.104	0.068	-0.017	-0.116

Gray indicates that autocorrelations in the precipitation time series were corrected.

#### Precipitation trend analysis

The trends of precipitation were analyzed using the M-K trend test, Spearman’s rho test and linear trend estimation. The significant (at the 5% confidence level) trends identified by two methods were italicized and underlined. Abrupt changes in the precipitation data were analyzed using the M-K test.

**Fig 2:**
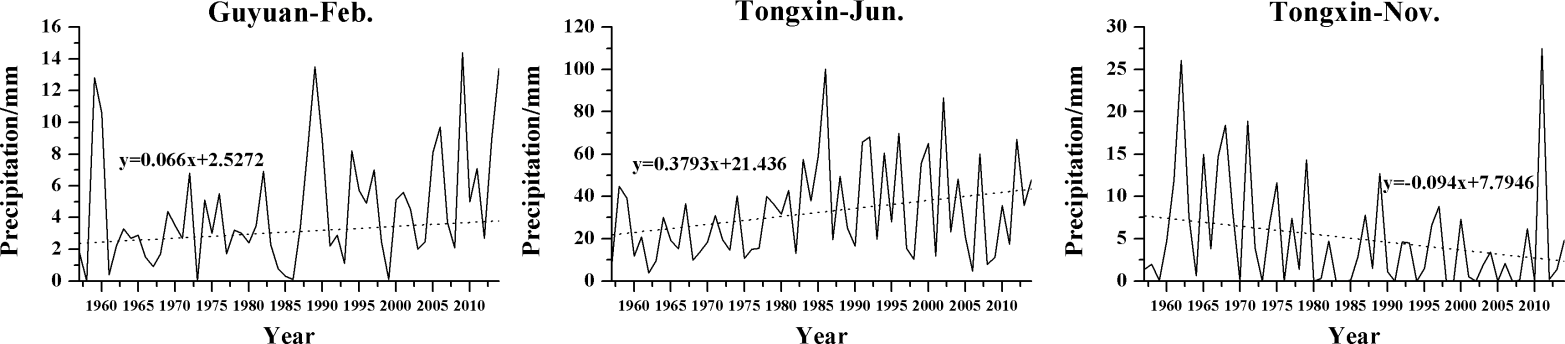
Variations in monthly precipitation at stations with significant trends during the study period.

The results of the analysis of monthly precipitation trends (Table 5) show that at the 11 synoptic stations, there was a decreasing trend in the precipitation of July, August and November, whereas both decreasing and increasing trends in the precipitation occurred of other months. The monthly precipitation for February at Guyuan and for June at Tongxin showed significant increasing trends with rates of 0.066 and 0.379 mm/year, respectively. The monthly precipitation for November at Tongxin showed a significant decreasing trend with a rate of 0.094 mm/year. The significant trends of the corresponding time series are shown in Fig 2.

**Table 5:** Results of the statistical tests for monthly precipitation data from 1957 to 2014.

Station name	Test	Jan.	Feb.	Mar.	Apr.	May	Jun.	Jul.	Aug.	Sep.	Oct.	Nov.	Dec.
**Lintao**	Z	-0.329	0.201	-0.168	0.423	-0.141	0.121	-1.167	-1.375	-1.167	-1.120	-1.436	-0.040
	S	-0.350	0.291	-0.300	0.427	-0.094	0.170	-1.242	-1.469	-1.404	-1.106	-1.456	-0.190
	b	-0.004	0.008	-0.045	-0.031	-0.007	0.034	-0.417	-0.712	-0.350	-0.128	-0.123	-0.001
**Linxia**	Z	0.550	1.912	1.308	-0.034	-0.812	1.308	-0.268	-0.107	-0.825	-0.349	-0.074	1.100
	S	0.810	1.740	1.204	-0.036	-0.699	1.336	-0.283	-0.111	-0.796	-0.387	-0.252	1.450
	b	0.023	0.051	0.074	0.026	-0.138	0.306	-0.075	-0.322	-0.159	-0.069	-0.026	0.015
**Huanxian**	Z	0.001	0.838	-1.241	-0.872	-0.651	1.831	-0.409	-0.570	-0.470	-0.275	-1.449	0.001
	S	-0.003	0.942	-1.114	-0.780	-0.662	1.865	-0.344	-0.547	-0.422	-0.331	-1.770	0.019
	b	0.020	0.011	-0.060	-0.062	-0.038	0.461	-0.034	-0.571	-0.112	-0.156	-0.051	0.003
**Yuzhong**	Z	0.121	1.449	0.054	-0.228	-0.114	1.436	-1.087	-1.744	-0.617	-0.456	-0.255	-0.812
	S	0.461	1.714	0.172	-0.246	-0.140	1.440	-1.107	-1.715	-0.650	-0.369	-0.610	-1.400
	b	0.008	0.048	-0.005	-0.070	0.006	0.344	-0.273	-0.695	-0.156	-0.084	-0.051	-0.012
**Guyuan**	Z	0.678	*2*.*569*	0.832	-0.570	-1.529	0.389	-1.389	-0.731	-0.470	0.329	-1.603	0.926
	S	0.980	*2*.*754*	0.893	-0.573	-1.644	0.500	-1.482	-0.632	-0.537	0.278	-2.030	1.250
	b	0.029	0.066	0.052	-0.032	-0.326	0.174	-0.453	-0.385	-0.214	-0.032	-0.070	0.013
**Tongxin**	Z	-0.476	0.020	-0.805	-1.684	-1.046	*2*.*220*	-0.342	-0.745	-0.463	-0.651	*-1*.*952*	-0.154
	S	-0.770	0.040	-0.662	-1.564	-0.828	*2*.*136*	-0.340	-0.780	-0.442	-0.587	*-2*.*600*	-0.360
	b	0.005	-0.006	-0.028	-0.114	-0.111	0.379	-0.055	-0.459	-0.037	-0.072	-0.094	-0.008
**Hequ**	Z	-1.107	-0.926	-1.234	-1.100	1.422	1.704	-1.328	-0.926	-0.288	-0.919	-0.678	-0.637
	S	-1.590	-1.080	-1.234	-1.023	1.719	1.826	-1.329	-0.947	-0.308	-0.846	-0.840	-0.860
	b	-0.048	-0.024	-0.042	-0.163	0.177	0.265	-0.532	-0.687	-0.209	-0.066	-0.025	-0.016
**Xingxian**	Z	-0.590	-0.181	-0.926	-0.322	1.228	1.731	-1.154	-0.832	0.208	-1.570	-0.309	-0.134
	S	-0.860	-0.061	-0.868	-0.153	1.312	1.908	-1.106	-0.878	0.078	-1.408	-0.145	-0.340
	b	-0.013	-0.020	-0.047	-0.047	0.202	0.563	-0.384	-0.657	-0.095	-0.130	0.021	0.025
**Suide**	Z	-0.711	0.181	-0.771	-1.248	-0.953	-0.490	-0.590	-1.207	0.335	-0.879	-1.020	-0.785
	S	-1.050	0.321	-0.809	-1.147	-0.916	-0.576	-0.620	-1.200	0.354	-0.883	-0.225	-1.090
	b	-0.002	-0.014	-0.035	-0.183	-0.243	-0.050	0.083	-0.642	0.106	-0.226	0.020	-0.010
**Wuqi**	Z	-1.060	0.932	-0.382	-1.764	-0.402	1.321	-0.067	-1.040	-0.001	-1.134	-0.939	0.315
	S	-0.697	0.824	0.275	-1.790	-0.416	1.441	-0.185	-0.876	-0.012	-1.138	-0.723	0.540
	b	-0.010	-0.006	-0.023	-0.245	-0.172	0.337	0.019	-0.431	-0.039	-0.337	-0.062	-0.003
**Yan’an**	Z	-0.201	1.469	-0.510	-1.046	-0.295	0.416	-0.859	-0.751	-0.409	-0.275	-1.107	-0.664
	S	-0.240	1.557	-0.515	-1.053	-0.302	0.408	-0.806	-0.753	-0.336	-0.254	-0.103	-0.890
	b	-0.003	0.055	-0.041	-0.139	-0.136	0.082	0.103	-0.417	-0.227	-0.090	-0.085	-0.014

Z: M-K trend test; S: Spearman’s rho test; b (mm/a): Slope of linear regression. *Italic type* represent trends identified by 2 statistical methods and trends that are statistically significant at the 5% level.

**Fig 3:**
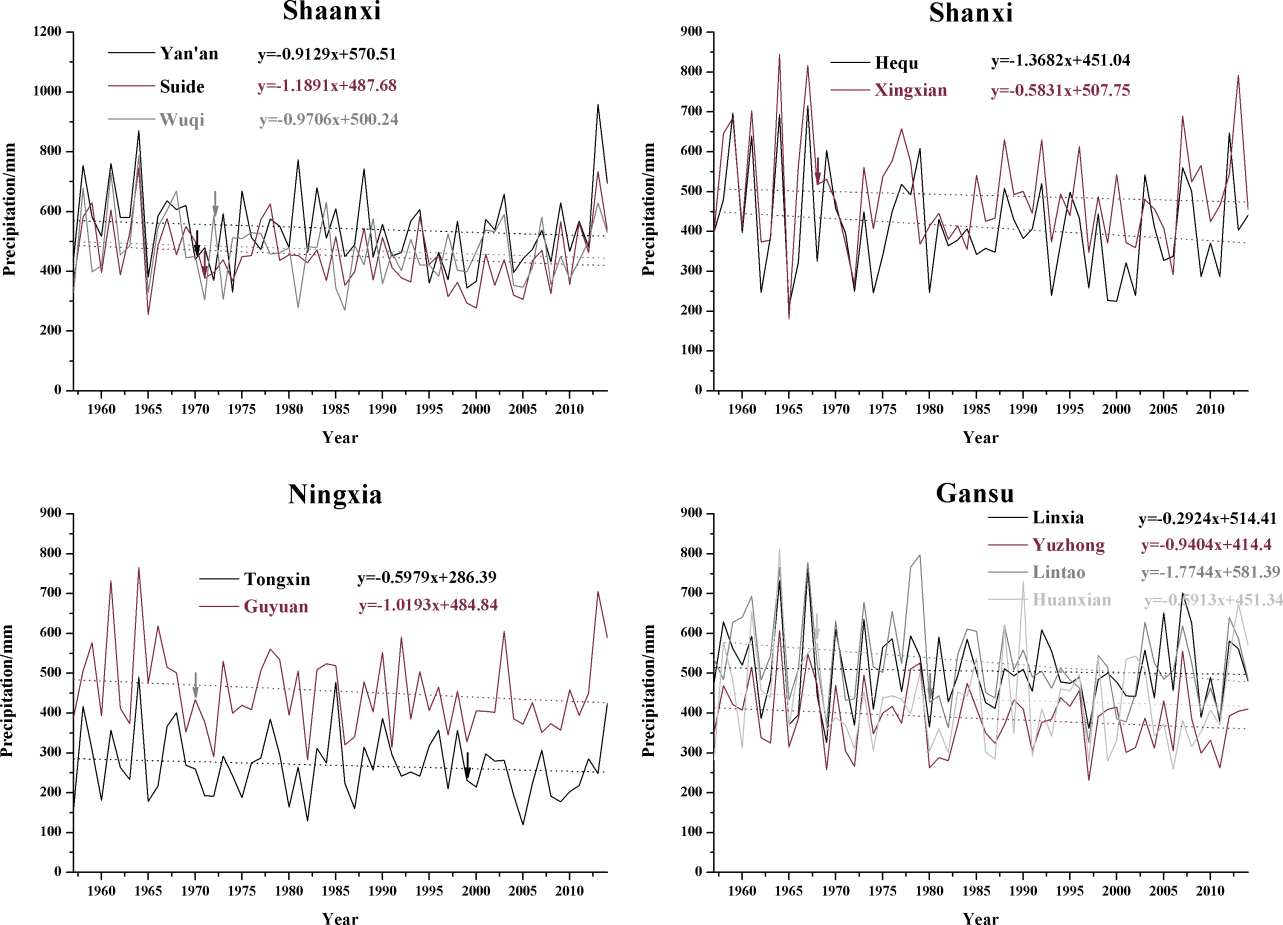
The annual precipitation time series at the 11 synoptic stations. The corresponding arrows indicate the time point of mutation of each station with more obvious abrupt change.

The annual precipitation time series at the 11 synoptic stations during the study period are shown in Fig 3. There were significant spatial differences and inter-annual variations in the precipitation at the synoptic stations. The annual mean precipitation varied between 268.7 mm (Tongxin) and 543.6 mm (Yan’an). The highest value appeared at Yan’an in 2013 (959.1 mm, which caused severe flooding in the local area), and the lowest annual value of 119.4 mm was detected at Tongxin in 2005. The results of the linear trend estimation for the annual precipitation time series (Fig 3) show that the annual precipitation decreased with fluctuations at the 11 synoptic stations. The largest decrease, 17.74 mm/decade, occurred at Lintao, and the smallest decrease, 2.92 mm/decade, occurred at Linxia.

The results of the analysis of trends in the annual and seasonal precipitation (Table 6) show that the summer and autumn precipitation had decreasing trends, whereas increasing and decreasing trends co-existed in the spring and winter precipitation at the 11 synoptic stations. In particular, winter precipitation markedly increased at Linxia and Guyuan, whereas a significant decreasing trend was seen at Hequ. All the test results for the annual precipitation showed decreasing trends to varying degrees. This is in agreement with the results of the linear trend estimations of the annual precipitation at the 11 synoptic stations (Fig 3) and indicates an overall drying trend in the study area between 1957 and 2014.

**Table 6:** Results of the statistical tests for the seasonal and annual precipitation data over the period from 1957 to 2014.

Station name	Test	Spring	Summer	Autumn	Winter	Annual
**Lintao**	Z	-0.215	-1.469	-1.469	-0.027	-1.597
	S	-0.222	-1.597	-1.503	-0.161	-1.779
	b	-0.083	-1.095	-0.601	0.001	-1.774
**Linxia**	Z	0.228	-0.228	-0.805	*2*.*428*	-0.651
	S	0.118	-0.229	-0.785	*2*.*460*	-0.620
	b	-0.039	-0.090	-0.253	0.092	-0.432
**Huanxian**	Z	-0.892	-0.268	-0.986	1.234	-0.550
	S	-0.411	-0.133	-0.889	1.279	-0.579
	b	-0.160	-0.145	-0.368	0.036	-0.591
**Yuzhong**	Z	0.107	-0.959	-0.879	1.523	-1.167
	S	0.044	-0.945	-0.945	1.517	-1.244
	b	-0.069	-0.624	-0.291	0.042	-0.940
**Guyuan**	Z	-1.422	-0.517	-0.624	*3*.*052*	-1.194
	S	-1.347	-0.461	-0.731	*3*.*278*	-1.179
	b	-0.306	-0.505	-0.314	0.099	-1.019
**Tongxin**	Z	-1.536	-0.027	-1.093	-0.188	-0.704
	S	-1.529	-0.008	-0.826	-0.193	-0.614
	b	-0.252	-0.135	-0.203	-0.005	-0.598
**Hequ**	Z	0.315	-1.020	-0.369	*-2*.*086*	-0.986
	S	0.355	-0.928	-0.345	*-2*.*087*	-1.004
	b	-0.027	-0.954	-0.300	-0.081	-1.368
**Xingxian**	Z	0.563	-0.986	-0.242	-0.463	-0.362
	S	0.475	-0.870	-0.220	-0.402	-0.408
	b	0.107	-0.478	-0.204	0.003	-0.583
**Suide**	Z	-1.160	-1.194	-0.067	-0.087	-1.764
	S	-1.337	-1.188	-0.005	-0.099	-1.545
	b	-0.455	-0.609	-0.100	-0.017	-1.189
**Wuqi**	Z	-1.603	-0.255	-0.295	0.329	-0.617
	S	-1.607	-0.166	-0.385	0.371	-0.692
	b	-0.439	-0.075	-0.438	-0.011	-0.971
**Yan’an**	Z	-0.657	-0.973	-0.577	1.221	-1.241
	S	-0.745	-0.937	-0.501	1.201	-1.299
	b	-0.316	-0.232	-0.402	0.045	-0.913

Z: M-K trend test; S: Spearman’s rho test; b (mm/a): Slope of linear regression. *Italic type* represent trends identified by 2 statistical methods that are statistically significant at the 5% level.

**Fig 4:**
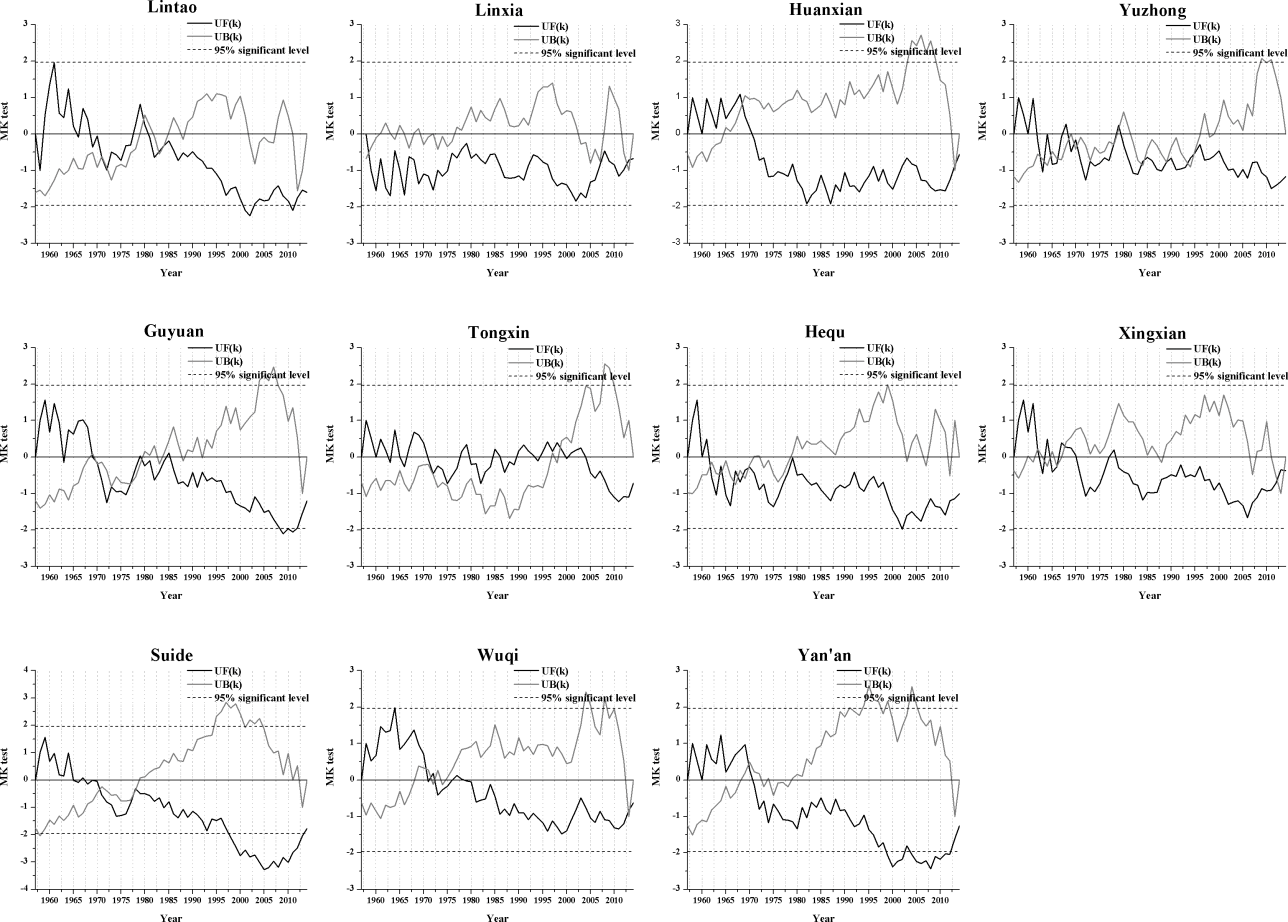
An analysis of the abrupt changes in the annual precipitation at the 11 synoptic stations for the period from 1957 to 2014.

The results of analyzing abrupt changes in the annual precipitation data (Fig 4) show that the decreasing trends began at different times during the study period at each station, but most of the meteorological stations experienced a mainly decreasing trend after the 1980s. Only Suide and Yan’an exhibited significant decreasing trends, and the significant decrease mainly falls between 1998 and 2013. Fig 4 also shows that the UF (k) and UB (k) curves for some meteorological stations intersect more than once, which makes it difficult to identify the specific year in which the precipitation abruptly changed. The stations with more obvious abrupt changes in precipitation were Lintao (around 1980), Huanxian (1968), Guyuan (1970) Tongxin (1999), Xingxian (1968), Suide (1971), Wuqi (around 1972) and Yan’an (1970), and they were marked in Fig 3 using the corresponding arrow.

### Temperature analysis

#### Temperature characteristics

Table 7 shows the characteristics of the annual mean temperature at the 11 synoptic stations between 1957 and 2014. During the study period, the annual mean temperature varied between 6.69 and 9.95°C. The lowest annual mean temperature was found at Guyuan (6.69°C), as was the largest CV (12.4%), which indicates a low temperature with poor stability. The annual mean temperature at Suide was 9.89°C, which was slightly lower than that at Yan’an and was the second lowest; however, the CV was relatively small (6.0%), and the high temperature remained stable.

**Table 7:** Statistical parameters for the annual temperature data from the 11 synoptic stations during the period from 1957 to 2014.

Station name	Min (°C)	Max/(°C)	Mean/(°C)	Standard deviation (°C)	CV (%)	Skewness	Kurtosis
**Lintao**	6.32	8.38	7.30	0.52	7.1	0.240	-0.452
**Linxia**	6.01	8.34	7.12	0.56	7.9	0.322	-0.600
**Huanxian**	6.48	10.35	8.81	0.81	9.2	-0.298	-0.031
**Yuzhong**	5.62	8.05	6.84	0.54	7.8	-0.060	-0.128
**Guyuan**	5.23	8.57	6.69	0.83	12.4	0.469	-0.493
**Tongxin**	7.24	10.64	9.06	0.78	8.6	0.152	-0.705
**Hequ**	6.50	9.67	8.35	0.74	8.9	-0.416	-0.376
**Xingxian**	7.45	10.42	8.87	0.77	8.7	0.373	-0.658
**Suide**	8.82	11.32	9.89	0.59	6.0	0.412	-0.213
**Wuqi**	6.84	9.20	8.03	0.55	6.8	-0.366	-0.556
**Yan’an**	8.42	11.47	9.95	0.78	7.9	0.353	-0.840

CV: Coefficient of variation.

#### Autocorrelation analysis of temperature series

As in the above autocorrelation analysis of precipitation series, we completely eliminated autocorrelation in the original temperature data (Table 8). The dark areas in the table indicate that the original temperature data exhibited autocorrelation, which was eliminated using TFPW.

**Table 8:** Lag-1 serial correlation coefficients of temperature on different scales.

Station name	Test	Spring	Summer	Autumn	Winter	Annual
**Lintao**	r_1_	0.262	0.355	0.237	0.282	0.591
	r_1_’	0.038	0.028	—	-0.099	0.012
**Linxia**	r_1_	0.389	0.567	0.152	0.274	0.633
	r_1_’	0.039	0.007	—	-0.144	0.038
**Huanxian**	r_1_	0.51	0.591	0.396	0.217	0.638
	r_1_’	0.005	-0.126	0.065	—	-0.009
**Yuzhong**	r_1_	0.263	0.367	0.155	0.164	0.506
	r_1_’	0.027	0.014	—	—	-0.002
**Guyuan**	r_1_	0.497	0.601	0.394	0.288	0.661
	r_1_’	0.003	-0.062	0.091	-0.055	0.024
**Tongxin**	r_1_	0.381	0.317	0.409	0.417	0.679
	r_1_’	0.066	0.02	0.006	-0.141	0.032
**Hequ**	r_1_	0.364	0.277	0.328	0.233	0.481
	r_1_’	0.054	-0.03	-0.056	—	-0.119
**Xingxian**	r_1_	0.349	0.271	0.194	0.345	0.557
	r_1_’	0.071	-0.016	—	-0.038	0.009
**Suide**	r_1_	0.322	0.094	-0.037	0.175	0.379
	r_1_’	0.033	—	—	—	0.009
**Wuqi**	r_1_	0.122	0.245	0.076	0.174	0.423
	r_1_’	—	0.239	—	—	0.006
**Yan’an**	r_1_	0.491	0.519	0.421	0.374	0.746
	r_1_’	-0.058	-0.013	-0.001	-0.203	-0.106

Gray indicates autocorrelation in the time series.

r_1_ indicates the lag-1 serial correlation coefficient of original temperature data

r_1_’ indicates the lag-1 serial correlation coefficient of the new series after TFPW.

#### Temperature trend analysis

**Fig 5:**
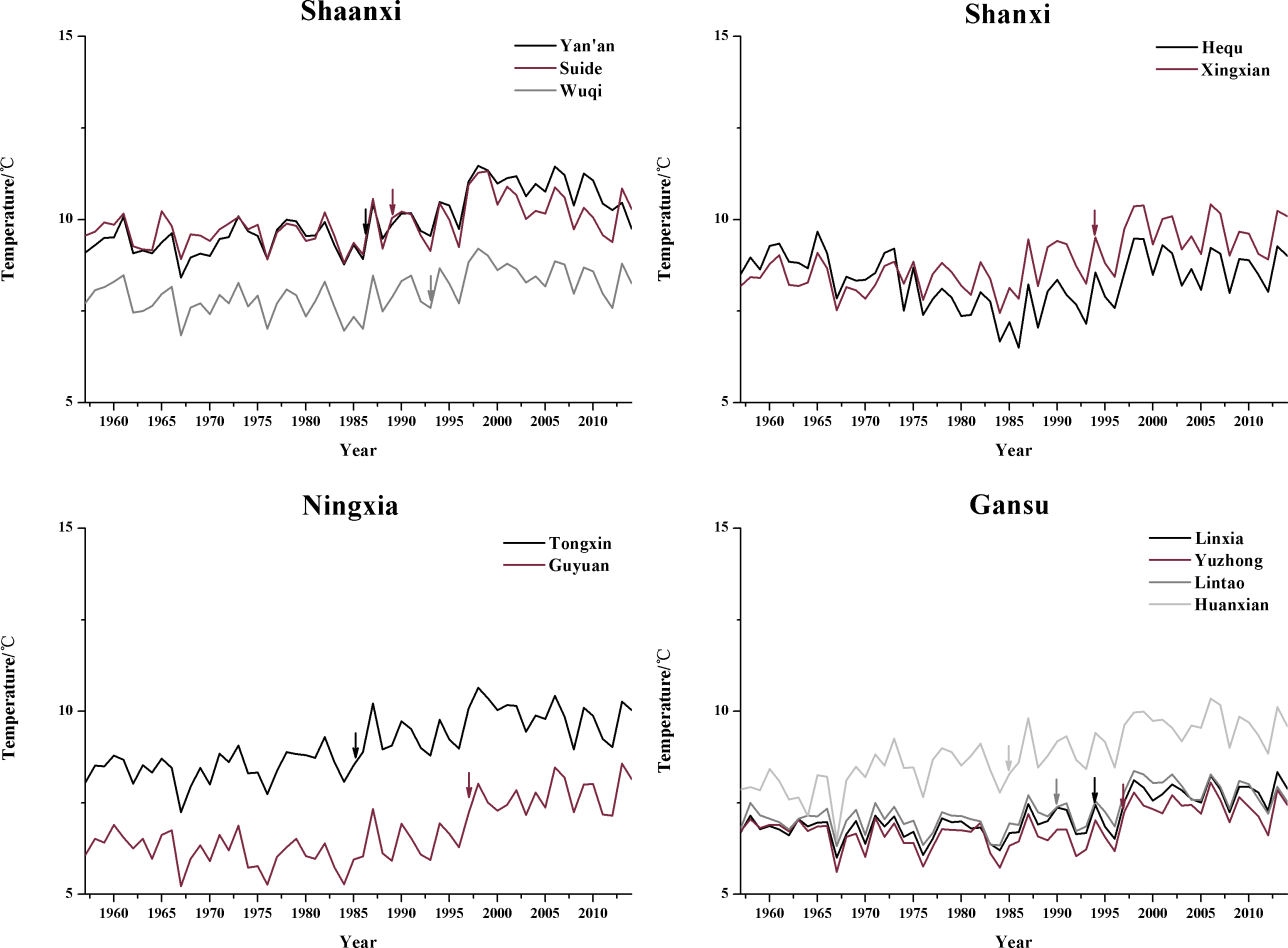
The annual temperature time series at the 11 synoptic stations. The corresponding arrows indicate the time point of mutation of each station.

Table 9 shows the statistical results of the trend tests for the seasonal and annual mean temperatures at the 11 synoptic stations for the period from 1957 to 2014. The autumn and winter temperatures showed downward trends only at Hequ. Except for the synoptic station at Hequ, the seasonal and annual mean temperatures showed upward trends. Hequ had the highest latitude among the synoptic stations within the study area. The different temperature trends might be related to latitude. As Fig 5 shows, the range of temperature variations was relatively small at the synoptic stations in the study area. Combined with the results of linear trend estimation (Table 9), we found that at almost all the synoptic stations, the annual mean temperature exhibited significant upward trends to varying degrees. The upward trend rate that was not significant, which occurred at Hequ, reached 0.01°C/decade. Combined with the results of the precipitation analysis in the previous section, we identified upward trends in the seasonal precipitation at a few synoptic stations, at which the temperature also showed significant upward trends. In arid and semiarid regions, a significant increase in temperature can counteract an increase in precipitation [30]. Therefore, the overall trend of drought and wetness in the study area was drying between 1957 and 2014.

**Table 9:** Statistical seasonal and annual temperature results for the period from 1957 to 2014.

Station name	Test	Spring	Summer	Autumn	Winter	Annual
**Lintao**	Z	1.778	1.912	*3*.*468*	2.096	*2*.*381*
	S	1.976	2.040	*4*.*042*	2.053	*2*.*768*
	b	0.012	0.01	0.017	0.015	0.009
**Linxia**	Z	*2*.*126*	*2*.*100*	*3*.*549*	*2*.*462*	*2*.*905*
	S	*2*.*236*	*2*.*298*	*3*.*866*	*2*.*682*	*3*.*345*
	b	0.015	0.012	0.018	0.018	0.012
**Huanxian**	Z	*3*.*347*	*2*.*287*	*3*.*750*	*3*.*159*	*3*.*133*
	S	*3*.*739*	*2*.*476*	*4*.*439*	*3*.*482*	*3*.*651*
	b	0.027	0.016	0.025	0.032	*0*.*018*
**Yuzhong**	Z	1.523	1.442	*3*.*354*	*2*.*482*	*2*.*146*
	S	1.613	1.520	*3*.*666*	*2*.*575*	*2*.*354*
	b	0.012	0.008	0.018	0.019	0.010
**Guyuan**	Z	*2*.*583*	*2*.*126*	*3*.*710*	*2*.*126*	*3*.*066*
	S	*2*.*862*	*2*.*391*	*4*.*423*	*2*.*232*	*3*.*518*
	b	0.022	0.012	0.025	0.024	0.017
**Tongxin**	Z	*3*.*200*	*2*.*113*	*3*.*790*	*2*.*073*	*3*.*133*
	S	*3*.*361*	*2*.*265*	*4*.*402*	*2*.*134*	*3*.*622*
	b	0.024	0.013	0.025	0.022	0.015
**Hequ**	Z	1.751	0.610	-1.241	-0.577	0.020
	S	1.918	0.575	-1.320	-0.601	0.113
	b	0.012	0.005	-0.011	-0.007	0.001
**Xingxian**	Z	*3*.*092*	1.912	*3*.*891*	*2*.*153*	*2*.*837*
	S	*3*.*393*	2.052	*4*.*514*	*2*.*252*	*3*.*329*
	b	0.021	0.015	0.026	0.025	0.017
**Suide**	Z	*2*.*784*	0.617	1.469	*1*.*918*	*2*.*140*
	S	*3*.*153*	0.904	1.518	*2*.*082*	*2*.*459*
	b	0.019	0.006	0.008	0.023	0.011
**Wuqi**	Z	*2*.*321*	1.664	*2*.*314*	*2*.*428*	*1*.*992*
	S	*2*.*408*	1.943	*2*.*453*	*2*.*660*	*2*.*294*
	b	0.015	0.010	0.013	0.024	0.010
**Yan’an**	Z	*3*.*092*	1.630	*3*.*119*	1.630	*2*.*247*
	S	*3*.*264*	1.644	*3*.*578*	1.736	*2*.*521*
	b	0.022	0.009	0.021	0.018	0.011

Z: M-K trend test; S: Spearman’s rho test; b (°C/a): Slope of linear regression. *Italic type* represent trends identified by 2 statistical methods that are significant at the 5% level.

**Fig 6:**
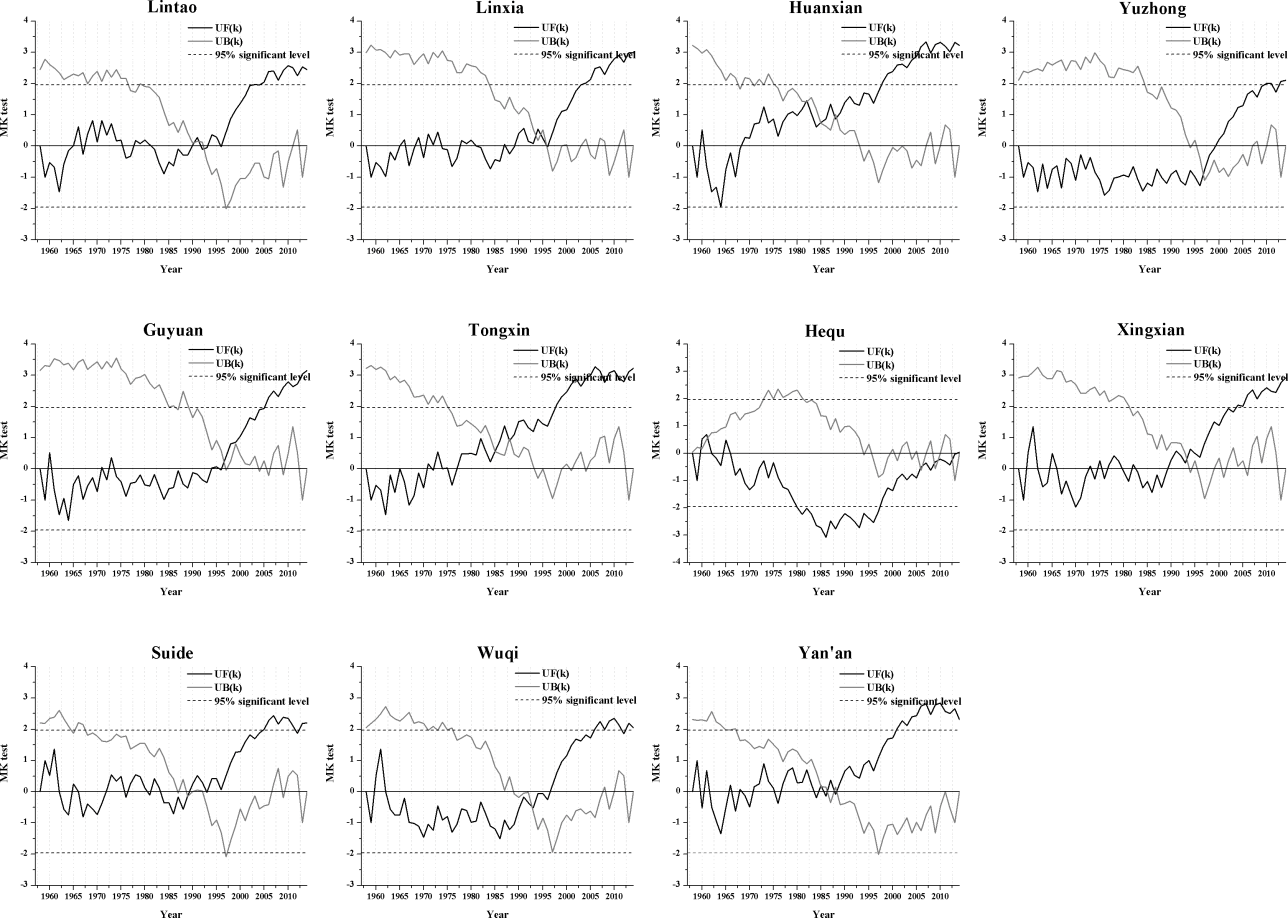
Abrupt changes in the annual temperature at the 11 synoptic stations during the period from 1957 to 2014.

Fig 6 shows the results of the test for abrupt changes in the annual mean temperature at the 11 synoptic stations (which used the same method as the analysis of abrupt changes in precipitation). The results show that the increasing trend at each station started at a different time during the study period, but most of the meteorological stations mainly experienced an increasing trend after 1990, and the increasing trend has become significant since 2005. The year of abrupt changes in temperature at each station is generally between 1985 and 1998. Time point of mutation were marked in Fig 5 using the corresponding arrow based on the Fig 6. Hequ station is a special example in the study area, the temperature can be obviously divided into three stages (1957–1980, the mean temperature was 8.77°C; 1981–1997, the mean temperature was 7.68°C; 1998–2014, the mean temperature was 8.80°C); the temperature of Hequ station almost showed a downward trend during the study period, which was significant from 1980 to 1997, and the trend began to shift to an upward trend in 2014.

### Morlet wavelet analysis

#### Wavelet analysis of precipitation

**Fig 7:**
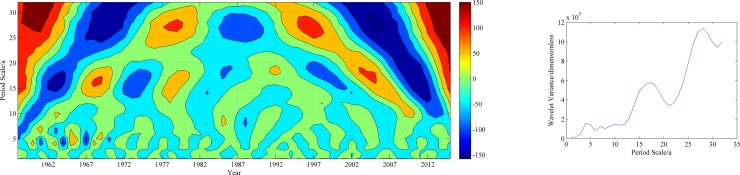
The isoline of the real parts of the Morlet wavelet coefficients and the wavelet variance on the principal scales of annual precipitation.

Fig 7 shows contour and variance maps of the real parts of the Morlet wavelet coefficients for the annual precipitation and the average annual mean precipitation in the study area. In the contour map, positive values indicate relatively high precipitation, and negative values indicate the opposite. The results clearly show the fluctuations of the real parts of the Morlet wavelet transform coefficients, as reflected by the alternating relatively high and low precipitation at the synoptic stations. Precipitation fluctuation centers with alternating positive and negative values were clearly observed on time scales of 7–8 years, 16–17 years and 27–28 years. That is, the precipitation significantly varied with above-normal and below-normal fluctuations within the calculation time domain.

The wavelet variance reflects the distribution of fluctuation energy over the scales. The wavelet variance map can be used to determine the major time scale, namely, the primary cycle in the precipitation time series. The wavelet variance maps of the mean precipitation in the study area (Fig 7) show main significant peaks at 27–28 years and 16–17 years. The highest peaks of the variance corresponded to a scale of 27–28 years, suggesting that 27–28 years was associated with the strongest cyclic fluctuations and therefore, was defined as the first primary cycle. The secondary cycle was mainly concentrated on a scale of 16–17 years.

**Fig 8:**
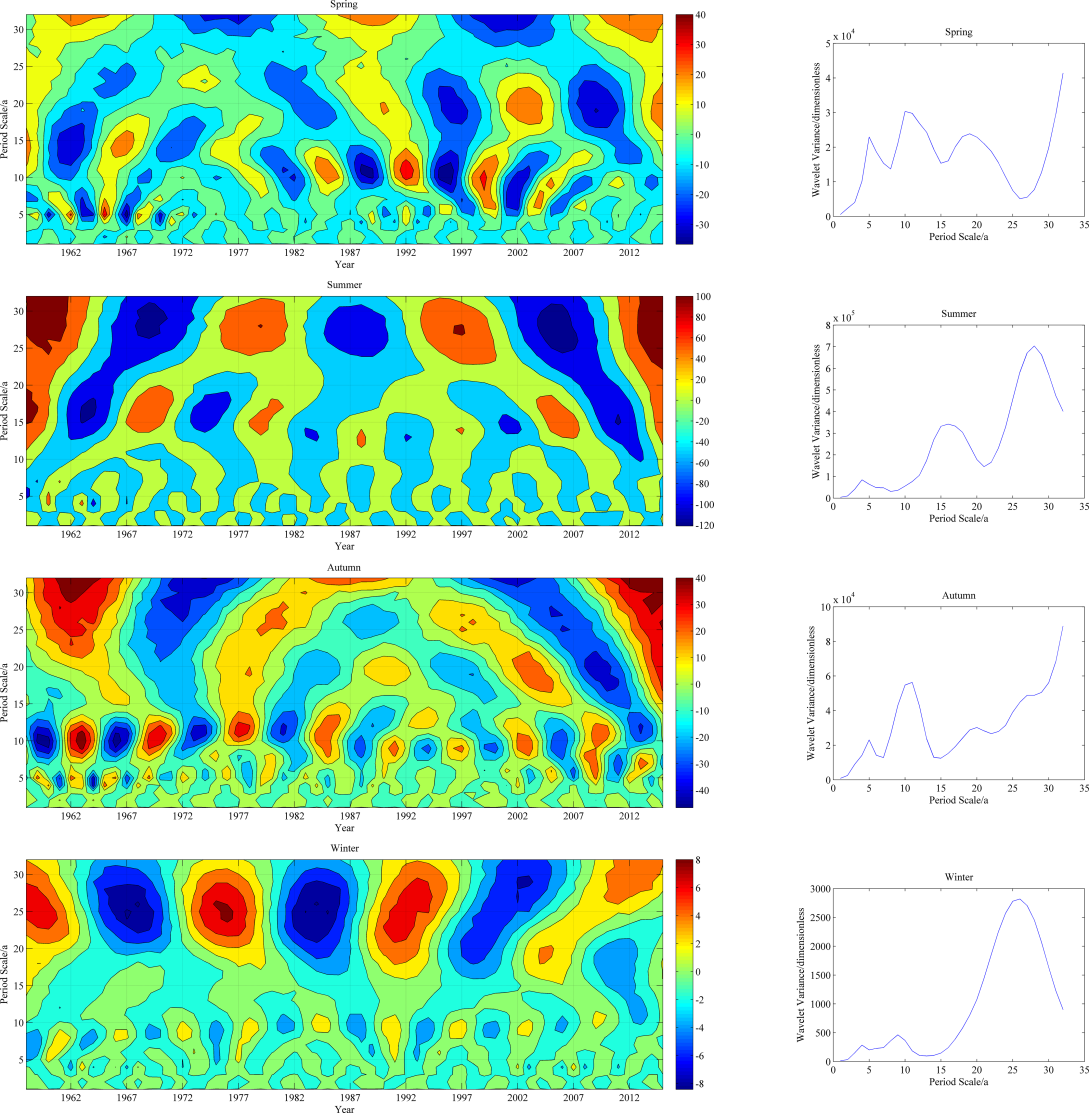
Isolines of the real parts of the Morlet wavelet coefficients and the wavelet variance for the principal scales of seasonal precipitation.

Fig 8 illustrates the contour and variance maps of the real parts of the Morlet wavelet coefficients for the seasonal mean precipitation in the study area. The precipitation significantly varied with above-normal and below-normal fluctuations in the spring, summer, autumn and winter on scales of 10 years, 28 years, 10 years and 26 years, respectively. These four periods were associated with the strongest cyclic fluctuations and therefore, were defined as the first primary cycles.

#### Wavelet analysis of temperature

**Fig 9:**
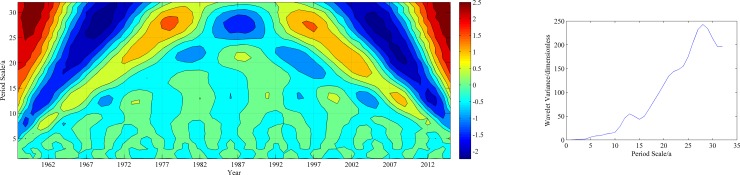
The isoline of the real parts of the Morlet wavelet coefficients and the wavelet variance for the principal scales of annual temperature.

Fig 9 depicts contour and variance maps of the real parts of the Morlet wavelet coefficients for the annual mean temperature and the average annual mean temperature in the study area. In the contour map, positive values indicate relatively high temperatures, and negative values indicate relatively low temperatures. The fluctuation characteristics reflect increases and decreases in temperature. A significant temperature fluctuation center was observed at approximately 28 years in the contour maps for the annual mean temperature in the study area. Another significant fluctuation center was seen at approximately 13 years. Combined with the wavelet variance maps in Fig 9, we concluded that 28 years was the first primary cycle and that 13 years was the second primary cycle for fluctuations in the annual mean temperature in the study area.

**Fig 10:**
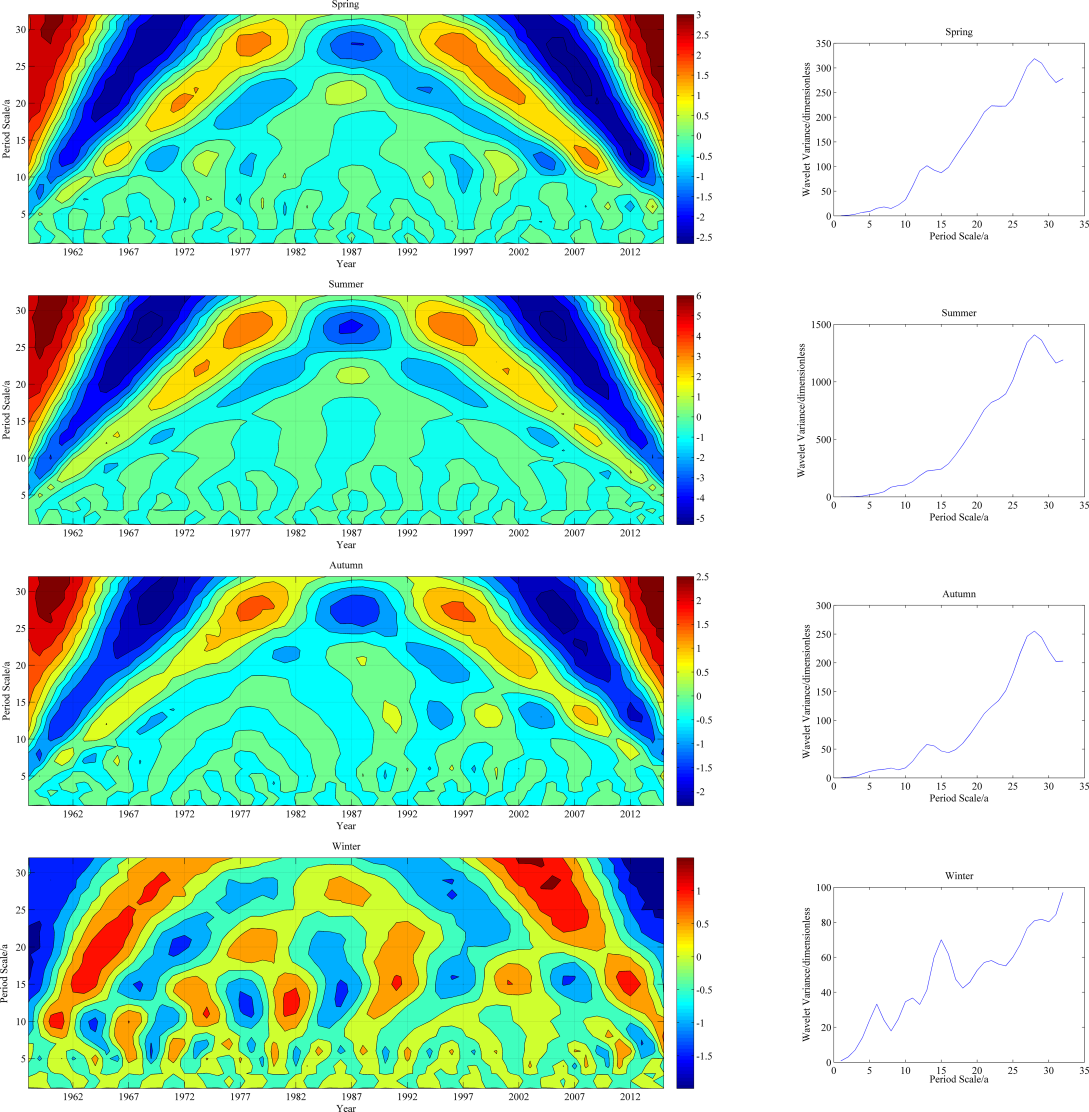
Isolines of the real parts of the Morlet wavelet coefficients and the wavelet variance for the principal scales of seasonal temperature.

Fig 10 presents contour and variance maps of the real parts of the Morlet wavelet coefficients for the seasonal mean temperature in the study area. The temperatures of the four seasons showed similar fluctuations in the study area, and the first primary cycle was approximately 28 years. Additionally, the winter temperature showed a strong cyclicality at approximately 15 years.

### SPEI analysis

Similar to the SPI, the SPEI can reflect agricultural drought conditions on a time scale of 1–6 months and, on a longer time scale (6 months or longer), can be used in research on hydrological drought in groundwater, rivers and reservoir reserves [9, 11, 31–32].

**Fig 11:**
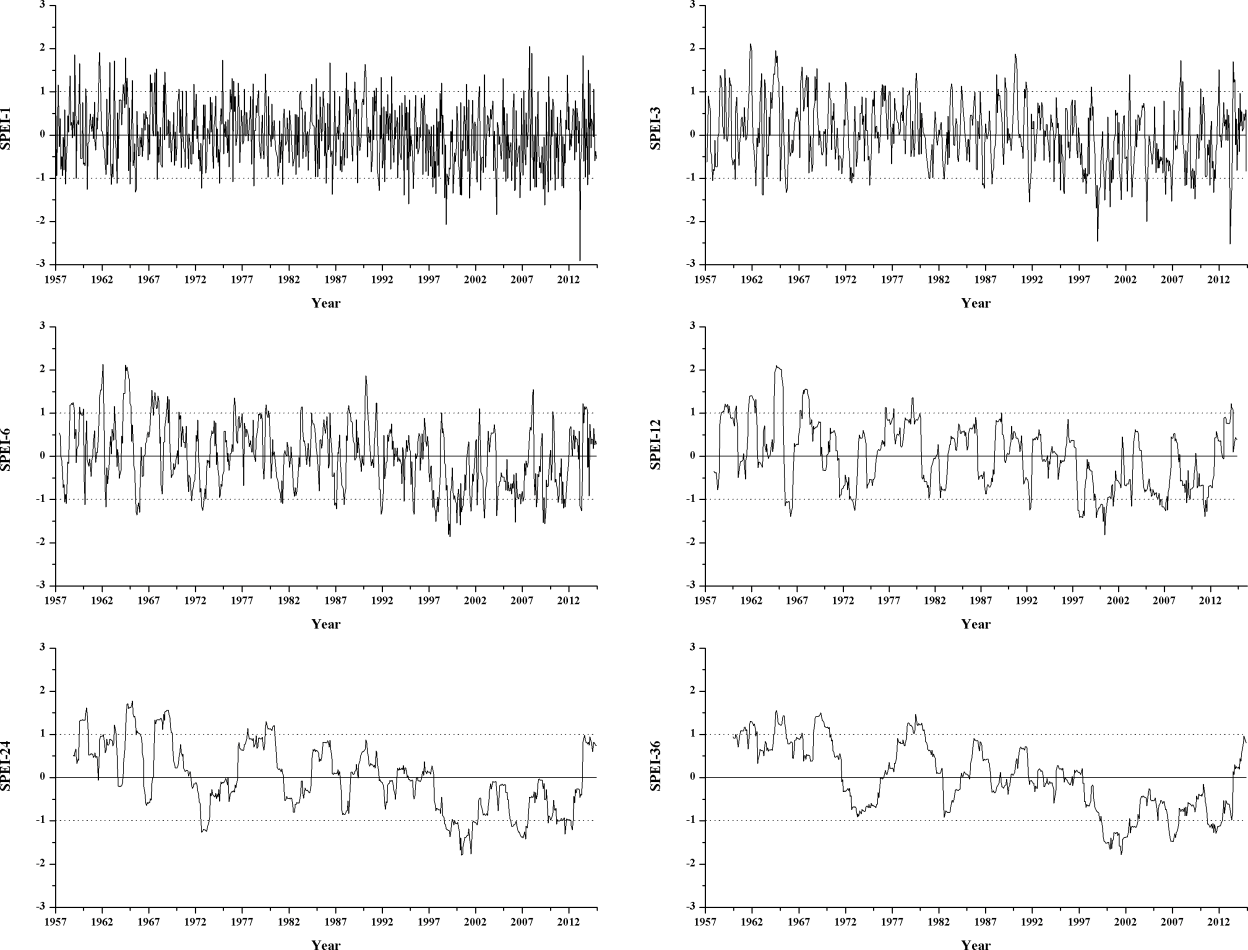
The SPEI for different time scales over the period from 1957 to 2014.

**Fig 12:**
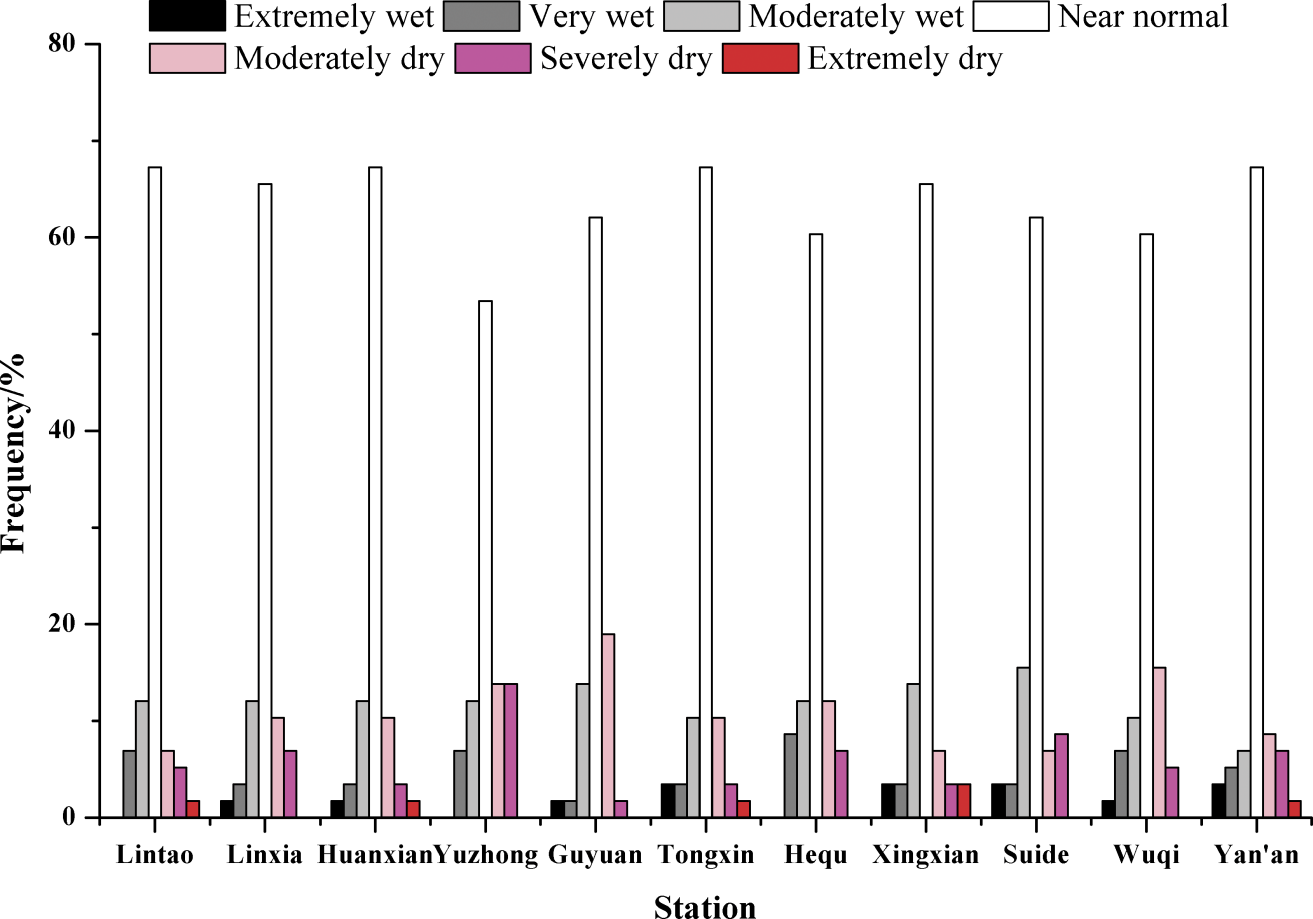
The distribution of dry, normal and wet years at the 11 synoptic stations during the period from 1957 to 2014.

The means of the SPEI on different time scales at the 11 synoptic stations can be approximated as a basis for analyzing the drought and wetness across the entire study area. Fig 11 shows the SPEI on different time scales (1, 3, 6, 12, 24 and 36 months). SPEI-1 was relatively sensitive to short-term changes in both temperature and precipitation; the values showed large fluctuations, which fully reflected the short-term characteristics of the study area with respect to impermanent droughts and frequent fluctuations. The periods with higher frequencies of seasonal drought (SPEI-3) and droughts that lasted 6 months (SPEI-6) and 1 year (SPEI-12) mainly began in the late 1990s. More significantly, the SPEI-24 and the SPEI-36 showed alternating drought and wetness in the study area over the period from 1957 to 2014. The major wet period was in the 1960s, and the dry period lasted for a long period from the late 20th century to 2012. Taking into account the percentage of dry and wet years at the synoptic stations over the study period (Fig 12) showed no obvious difference in the frequency of dry and wet years in the study area between 1957 and 2014. Normal years accounted for more than 53.4% at all the stations and exceeded 60% at the majority of the stations. However, drought and wetness occurred in relatively concentrated periods, and persistent drought was dominant in the new century.

The values of the SPEI for each year and season at the 11 synoptic stations were analyzed using the M-K trend test. To analyze seasonal dry-wet changes, the values of the SPEI-3 corresponding to May, August, November and February were chosen to represent the four seasons. The values of the SPEI-12 corresponding to December were used as the basis for analyzing annual dry-wet changes. The results for the statistical variable *Z* are shown in Table 10. During the study period, only a few synoptic stations showed wetting trends of varying degrees in winter (Z > 0). At the remaining stations, both the seasonal and the annual values of the SPEI showed decreasing trends. The decreasing trends in the annual SPEI passed the significance test at 90% in all cases, indicating that the climate was becoming progressively drier in the study area. This is consistent with the results presented in the previous section, and the severity of the drought increased.

**Table 10:** The M-K trend test for the SPEI.

Season	Lintao	Linxia	Huanxian	Yuzhong	Guyuan	Tongxin	Hequ	Xingxian	Suide	Wuqi	Yan’an
**Spring**	-0.90	-0.92	-2.86^***^	-0.50	-2.83^***^	-2.95^***^	-1.33^*^	-1.60^*^	-1.85^**^	-2.03^**^	-2.47^***^
**Summer**	-1.77^**^	-0.67	-0.95	-1.23	-1.58^*^	-0.52	-1.02	-1.41^*^	-1.15	-0.55	-1.77^**^
**Autumn**	-2.03^**^	-1.45^*^	-1.61^*^	-1.70^**^	-1.34^*^	-2.43^***^	-0.35	-1.17	-0.28	-0.91	-1.33^*^
**Winter**	-0.38	2.32^***^	0.94	1.38^*^	3.12^***^	-0.45	-2.07^**^	-0.46	-0.72	0.23	0.72
**Annual**	-2.72^***^	-1.72^**^	-2.07^**^	-2.09^**^	-2.79^***^	-2.43^***^	-1.32^*^	-1.49^*^	-1.91^**^	-1.38^*^	-2.76^***^

*10% confidence level

**5% confidence level

***1% confidence level.

**Fig 13:**
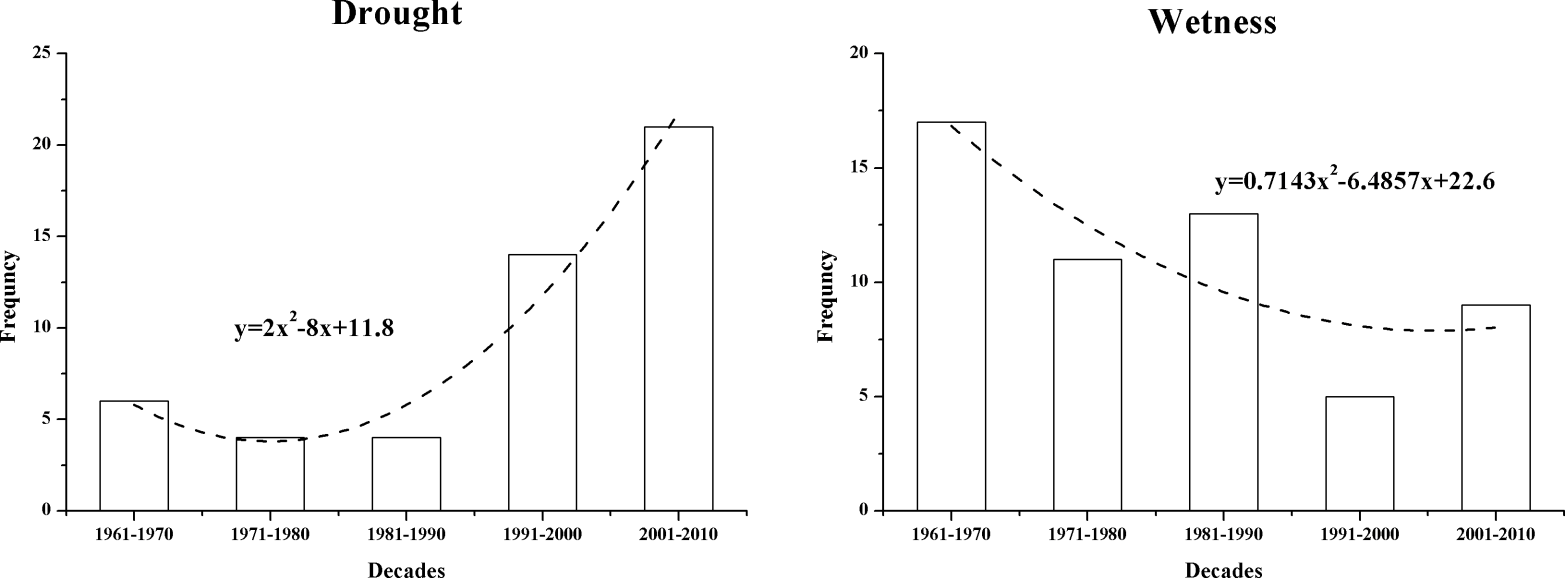
Drought and wetness frequencies based on the SPEI-1.

Fig 13 illustrates the frequencies of drought and wet events in different years based on the SPEI-1. The frequency of dry months markedly increased from the 1960s to the present, which contrasts with the decreasing trend in the wet months. This also indicates that the study area followed a trend toward drought with increasing severity.

## Discussion and conclusions

The loess hilly-gully region is one of the regions that suffers from the most serious soil erosion and drought in China and even in the world, and there was still a drying trend and the severity of the drought was increasing in this region. However, since the implementation of the “Grain for Green” policy, the precipitation and temperature in this region have improved to varying levels, the drier-and-hotter trend has been slightly restrained over the past years [33–34], it can be seen that vegetation restoration may improve the environmental conditions to a certain extent.

Soil moisture is a direct source of water required for plant growth [35] and a major limiting factor for plant growth and vegetation construction in arid and semiarid regions [6]. Precipitation and temperature affect the growth of vegetation together [36]. Atmospheric precipitation is the only way to recharge soil moisture on the Loess Plateau. Changes in soil moisture content are directly related to atmospheric precipitation [37]. The level of temperature influences water evapotranspiration and its role in drought formation [30], and soil moisture changes cannot be ignored. Therefore, it is more convincing to include temperature in an analysis of drought and wetness characteristics in this region, and the results are more reliable. This is the idea behind the SPEI, as used in the present study, which decreases the disadvantages of the SPI.

In this study, the monthly mean precipitation ranged from 22.4 to 45.3 mm, and the fluctuation in the monthly precipitation was intense (CV > 100%) at all stations. The main reason for this was that the fractured topography of the loess hilly-gully region of China formed microenvironments [38]. During the study period, the monthly precipitation for July, August and November showed decreasing trends, whereas the precipitation in the other months showed both decreasing and increasing trends at the 11 synoptic stations in the study area. The seasonal precipitation in summer and autumn exhibited decreasing trends, whereas the spring and winter precipitation showed both decreasing and increasing trends. In particular, there were significant increasing trends in winter precipitation at Linxia and Guyuan, in contrast to the significant decreasing trend observed at Hequ. The annual precipitation decreased with fluctuations; the largest decrease occurred at Lintao (17.74 mm/decade), and the smallest decrease occurred at Linxia (2.92 mm/decade). At several stations, the annual precipitation changes could be regarded as abrupt. During the study period, the annual mean temperature varied between 6.69 and 9.95°C. Except for the downward trends of the autumn and winter mean temperatures at Hequ, the seasonal and annual mean temperatures at the stations showed upward trends, including highly significant upward trends. At most stations, the temperature changes could be regarded as abrupt, and the abrupt temperature changes at these stations were mainly concentrated between 1985 and 1998. Hequ station is a special example in the study area, the temperature had an obvious low temperature period (1981–1997, the mean temperature was 7.68°C); the temperature at Hequ station almost showed a downward trend during the study period and the trend began to shift to upward in 2014, which reflect the different warming process with other stations [39]. Temperature and precipitation can be reliably predicted by analyzing the fluctuation cycle. In this study, the annual precipitation and annual mean temperatures showed significant cyclic fluctuations at the 11 synoptic stations in the study area; their first primary cycle was approximately 28 years. The seasonal temperatures and precipitation showed different cycle lengths; the cycles of spring, summer, autumn and winter precipitation were 10 years, 28 years, 10 years and 26 years long, respectively, and the fluctuation cycle of the seasonal temperatures was approximately 28 years long.

**Fig 14:**
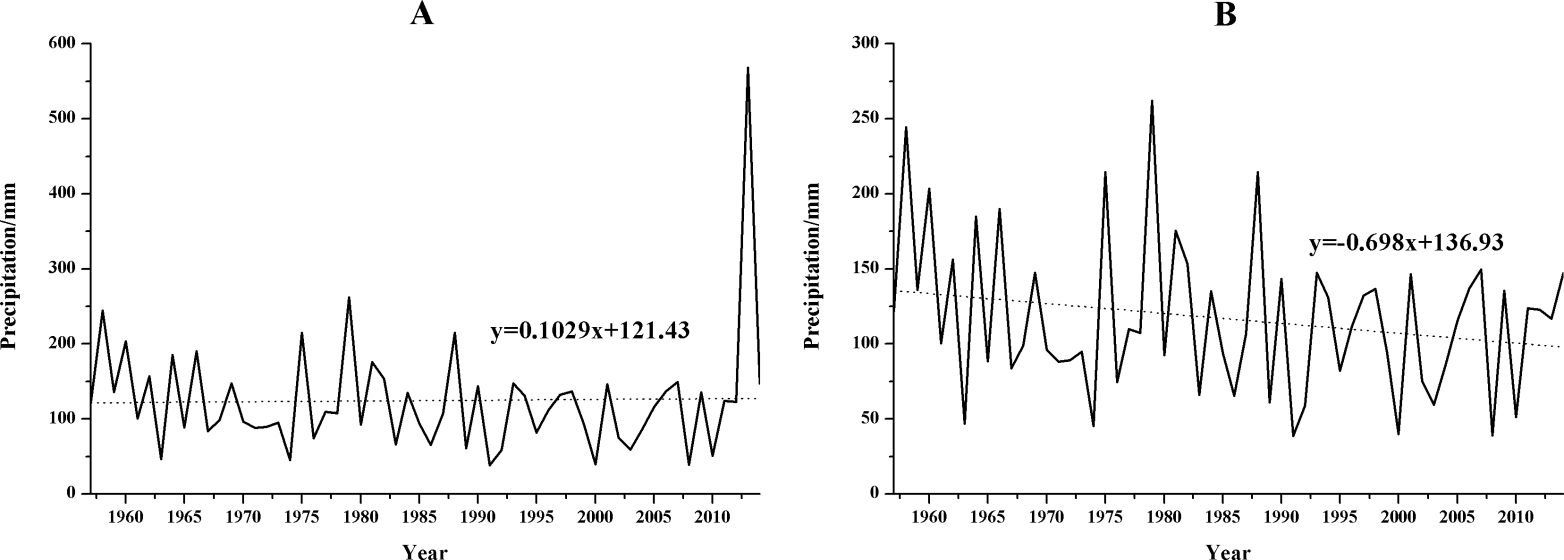
The precipitation at Yan’an station in July.

In the precipitation trend analysis (Table 4), the results of the linear trend estimations were occasionally different from those of the M-K trend test and Spearman’s rho test. Anomaly detection using a data processing system (DPS) revealed the presence of anomalies in the monthly precipitation with different results over the period from 1957 to 2014, which affected the estimation of the linear trends. The July precipitation at Yan’an (Z = −0.859, S = −0.806, b = 0.103) was analyzed as an example (Fig 14A). In July 2013, the measured precipitation at Yan’an was 568 mm, which was far more than the precipitation over the same period in other years. After excluding the anomaly, an approximate time series was obtained (Fig 14B). The results of the linear trend estimation after excluding the anomaly were consistent with the results of the M-K trend test and Spearman’s rho test. The presence of anomalies affects the results, whereas appropriate processing of the anomalies improves the accuracy of the statistical analysis. This reflects the fact that multiple analytical methods can be used in combination to improve the reliability of the results.

The applicability of the SPEI in China has been verified [40–41], which indicates that the SPEI can represent features and drought characteristics on multiple time scales in the loess hilly-gully region; a correlation between the SPEI and soil moisture has also been found [42]. The results of the SPEI analyses on different time scales showed that alternating drought and wetness occurred in the study area. The wet period was mainly in the 1960s, and the dry period occurred from the late 20th century to 2012. The numbers of dry and wet years were comparable, and the percentages of normal years were the highest (> 60% at the vast majority of synoptic stations). However, there was still a drying trend in the study area, and the severity of the drought was increasing. Previous studies have indicated that droughts occur more frequently and that their severity is increasing [43–46]. The same phenomenon occurred in this region, which may be because the regional topography is complex and diverse, affecting the precipitation and temperature distributions.

The analytical methods used in this study can be extended for use in climate change research in other regions, particularly arid and semiarid regions, and could provide some new methods for analyzing climatic characteristics and assessing droughts and wet/dry episodes. Research on the theory and technology of vegetation restoration at difficult sites is likely to be the focus of our next study. However, with the background of global warming, a dry soil layer comprising a surface dry soil layer and a subsurface dry soil layer may form in this region in response to drought and plant growth [47] and utilization. Researchers have investigated the subsurface dry soil layer [48–50]. The surface dry soil layer can affect the germination of plant seeds and the survival of shallow-rooted plants, thereby influencing the natural regeneration of vegetation. The characteristics and formation mechanisms of and abatement technologies for the surface dry soil layer need to be further studied to improve water use efficiency and facilitate vegetation construction and restoration in this region.

## Supporting information

S1 File The precipitation data used in this article. (XLSX)

S2 File The temperature data used in this article. (XLSX)

S3 File The SPEI data used in this article. (XLSX)

## References

[1] Wang LN, Zhu QK, Zhao WJ, Zhao XK. The drought trend and its relationship with rainfall intensity in the Loess Plateau of China. Natural Hazards. 2015; 77(1): 479–495.

[2] Wei J, Zhou J, Tian JL, He XB, Tang KL. Decoupling soil erosion and human activities on the Chinese Loess Plateau in the 20th century. Catena. 2006; 68: 10–15.

[3] Fu C, Wang YR. Response of monthly precipitation in the Loess Plateau in China to global climate change. Arid Zone Research. 2008; 25: 447–451.

[4] Liu YG. Analysis on the change trend of precipitation in north Shaanxi Province in the Loess Plateau. Arid Zone Research. 2007; 24: 49–55.

[5] Zheng BM, Tian CH, Wang Y, Guo YM. The theory and practice of small watershed dam system construction in the first deputy district of loess hilly-gully region. The yellow river press, Zhengzhou, 2004.

[6] Zhu QK, Zhang Y, Zhao LL, Qin W, Liu ZQ. Near natural vegetation restoration and afforestation in Loess Plateau of Northern Shaanxi, China. Science press, Beijing, 2012.

[7] Ficklin DL, Letsinger SL, Gholizadeh H, Maxwell JT. Incorporation of the Penman-Monteith potential evapotranspiration method into a Palmer Drought Severity Index Tool. Computers & Geosciences. 2015; 85: 136–141.

[8] He Y, Ye JY, Yang XY. Analysis of the spatio-temporal patterns of dry and wet conditions in the Huai River Basin using the standardized precipitation index. Atmospheric Research. 2015; 166: 120–128.

[9] Stagge JH, Tallaksen LM, Gudmundsson L, Van Loon AF, Stahl K. Candidate distributions for climatological drought indices (SPI and SPEI). International Journal of Climatology. 2015; 35: 4027–4040.

[10] Palmer WC. Meteorological droughts. U.S. Department of commerce weather bureau research paper. 1965; 45: 58.

[11] McKee TB, Doesken NJ, Kleist J. The relationship of drought frequency and duration to time scales. Proceedings of the 8th Conference on Applied Climatology, Anaheim, CA, USA. 1993.

[12] Vicente-Serrano SM, Beguería S, López-Moreno JI. A multi-scalar drought index sensitive to global warming: The standardized precipitation evapotranspiration index. Journal of Climate. 2010; 23: 1696–1718.

[13] Zhang BQ, Wu PT, Zhao XN, Gao XD. Spatiotemporal analysis of climate variability (1971–2010) in spring and summer on the Loess Plateau, China. Hydrological Process. 2014; 28: 1689–1702.

[14] Zhao WJ, Yu XY, Ma H, Zhu QK, Zhang Y, Qin W, et al. Analysis of precipitation characteristics during 1957–2012 in the semi-arid Loess Plateau, China. PLoS One. 2015; 10: e0141662. doi: 10.1371/journal.pone.0141662 26528917 PMC4631353

[15] Thornthwaite CW. An approach toward a rational classification of climate. Geography Review. 1948; 38: 55–94.

[16] National Weather Service. Climate prediction center. Available online at http://www.cpc.ncep.noaa.gov/.

[17] National climate center, Chinese academy of meteorological science, China meteorological administration, 2006.GB/T 20481–2006: Classification of meteorological drought. Standards press of China, Beijing.

[18] Yue S, Pilon P, Phinney B, Cavadias G. The influence of autocorrelation on the ability to detect trend in hydrological series. Hydrological Processes. 2002; 16: 1807–1829.

[19] Theil H. A rank invariant method of linear and polynomial regression analysis, part 3. Koninklijke Nederlandse Akademie van Wetenschappen. 1950; 53: 1397–1412.

[20] Sen PK. Estimates of the regression coefficients based on Kendall’s tau. Journal of the American Statistical Association. 1968; 63: 1379–1389.

[21] Sales JD, Delleur JW, Yevjevich VM, Lane WI. Applied modeling of hydrologic time series. Littleton, Colorado, USA: Water Resources Publication, 1980.

[22] Mann HB. Non-parametric tests against trend. Econometrica. 1945; 13: 245–259.

[23] Kendall MG. Rank correlation measures. Charles Griffin, London, UK, 1975.

[24] Lehmann EL. Nonparametrics: Statistical methods based on ranks. Holden-Day, San Francisco, 1975.

[25] Sneyers R. On the statistical analysis of series of observations. World Meteorological Organization, Technical Note No. 143, WMO No. 415, 1990.

[26] Sen AK, Ogrin D. Analysis of monthly, winter, and annual temperatures in Zagreb, Croatia, from 1864 to 2010: the 7.7-year cycle and the North Atlantic Oscillation. Theoretical and Applied Climatology. 2016; 123: 733–739.

[27] Cochran F, Brunsell N, Cabalzar A, van der Veld PJ, Azevedo E, Azevedo R, et al. Indigenous ecological calendars define scales for climate change and sustainability assessments. Sustainability Science. 2016; 11: 69–89.

[28] Huang HP, Han YP, Cao MM, Song JX, Xiao H. Spatial-temporal variation of aridity index of China during 1960–2013. Advances in Meteorology. 2016; 1536135.

[29] Storch HV, Navarra A. Analysis of climate variability − Applications of statistical techniques. New York: Springer-Verlag, 1995.

[30] Zavaleta ES, Thomas BD, Chiariello NR, Asner GP, Shaw MR, Field CB. Plants reverse warming effect on ecosystem water balance. Proceedings of the National Academy of Sciences of the United States of America. 2003; 100: 9892–9893. doi: 10.1073/pnas.1732012100 12907704 PMC187878

[31] Svoboda M, Hayes M, Wood D. Standardized precipitation index user guide. World Meteorological Organization (WMO-No.1090), Geneva, 2012.

[32] Li BQ, Zhou W, Zhao YY, Ju Q, Yu ZB, Liang ZM, et al. Using the SPEI to assess recent climate change in the Yarlung Zangbo River basin, south Tibet. Water. 2015; 7: 5474–5486.

[33] Gao P, Jiang GT, Wei YP, Mu XM, Wang F, Zhao GJ, et al. Streamflow regimes of the Yanhe River under climate and land use change, Loess Plateau, China. Hydrological Processes. 2015; 29(10): 2402–2413.

[34] Bai JJ, Di LP, Bai JT. NDVI and regional climate variation since the implementation of revegetation program in Northern Shaanxi Province, China. IEEE Journal of Selected Topics in Applied Earth Observations and Remote Sensing. 2041; 7(11): 4581–4588.

[35] Martinez-Fernandez J, Ceballos A. Temporal stability of soil moisture in a large-field experiment in Spain. Soil Science Society of America Journal. 2003; 67: 1647–1656.

[36] He JS, Xu JC, Gang CC, Li W, Luo RM, Peng CH. Critical climate periods for grassland productivity on China's Loess Plateau. Agricultural and Forest Meteorology. 2017; 233: 101–109.

[37] Yao XL, Fu BJ, Lü YH. Spatial patterns of soil moisture at transect scale in the Loess Plateau of China. Acta Ecologica Sinica. 2012; 32(16): 4961–4968.

[38] Sun ZH, Wang ZL, Cao XM, Yang Q, Liu ZC, Lei YP. Characteristics of drought change in the Loess Plateau area of Shaanxi based on the standardized precipitation index during 1971–2010. Journal of Desert Research. 2013; 33: 1560–1567.

[39] Han CH, Hao ZX, Zheng JY. Regionalization of temperature changes in China and characteristics of temperature in different regions during 1951–2010. Progress in Geography. 2013; 32(6): 887–896.

[40] Zhuang SW, Zuo HC, Ren PC, Xiong GJ, Li BD, Dong WC, et al. Application of standardized precipitation evapotranspiration index in China. Climatic and Environmental Research. 2013; 18(5): 617–625.

[41] Wang L, Chen W. Applicability analysis of standardized precipitation evapotranspiration index in drought monitoring in China. Plateau Meteorology. 2014; 33(2): 423–431.

[42] Zhao XK, Li ZY, Zhu QK. Response of soil moisture on climate characteristics based on SPI and SPEI in loess region of Northern Shaanxi. Transactions of the Chinese Society for Agricultural Machinery. 2016; 47(8): 155–163.

[43] Breshears DD, Cobb NS, Rich PM, Price KP, Allen CD, Balice RG, et al. Regional vegetation die-off in response to global-change-type drought. Proceedings of the National Academy of Sciences of the United States of America. 2005; 102: 15144–15148. doi: 10.1073/pnas.0505734102 16217022 PMC1250231

[44] Brown K. Environmental data.Water scarcity: Forecasting the future with spotty data. Science. 2002; 297: 926–927. doi: 10.1126/science.297.5583.926 12169710

[45] Easterling DR, Meehl GA, Parmesan C, Changnon SA, Karl TR, Mearns LO. Climate extremes: observations, modeling, and impacts. Science. 2000; 289, 2068–2074. 11000103 10.1126/science.289.5487.2068

[46] IPCC. Climate Change 2013: The physical science basis: contribution of working group I to the fifth assessment report of the intergovernmental panel on climate change. IPCC, 2013.

[47] Xie J, Chen JQ, Sun G, Zha TS, Yang B, Chu HS, et al. Ten-year variability in ecosystem water use efficiency in anoak-dominated temperate forest under a warming climate. Agricultural & Forest Meteorology. 2016; 218: 209–217.

[48] Jia XX, Shao MA, Zhang CC, Zhao CL. Regional temporal persistence of dried soil layer along south–north transect of the Loess Plateau, China. Journal of Hydrology. 2015; 528: 152–160.

[49] Yan WM, Deng L, Zhong YQW, Shangguan ZP. The characters of dry soil layer on the Loess Plateau in China and their influencing factors. PLoS One. 2015; 10: e0134902. doi: 10.1371/journal.pone.0134902 26241046 PMC4524687

[50] Jia YH, Shao MA. Dynamics of deep soil moisture in response to vegetational restoration on the Loess Plateau of China. Journal of Hydrology. 2014; 519: 523–531.

